# Ecological Momentary Assessment of Parental Well-Being and Time Use: Mixed Methods Compliance and Feasibility Study

**DOI:** 10.2196/67451

**Published:** 2025-04-23

**Authors:** Laura Altweck, Samuel Tomczyk

**Affiliations:** 1 Department Health and Prevention Institute of Psychology Universität Greifswald Greifswald Germany; 2 German Center for Child and Adolescent Health (DZKJ), partner site Greifswald/Rostock Greifswald Germany

**Keywords:** ecological momentary assessment, parents, feasibility, compliance, time use, well-being, stress, EMA, mixed methods, daily life, parents, online questionnaires, questionnaires, surveys, quantitative, qualitative, sociodemographic, mobile phone

## Abstract

**Background:**

Parents often juggle multiple conflicting responsibilities, including work, childcare, and the household, making them a particularly burdened group. However, the impact of daily routines and associated (poor) well-being among parents has received relatively little attention. Ecological Momentary Assessment (EMA) is increasingly being used to capture real-time data and can help address this research gap.

**Objective:**

This study aims to examine compliance rates and the feasibility of EMA for measuring daily well-being and time use among parents.

**Methods:**

An exploratory mixed-methods study was conducted with 74 German parents (57/74, 77% women, (age: mean 37.6, SD 5.9 years). Participants completed a baseline questionnaire, followed by 4 daily EMA surveys (at 7:30 AM, 12 PM, 16:30 PM, and 21:30 PM) over a 1-week period, and a follow-up questionnaire. A subset of parents was also subsequently interviewed. Sociodemographic background and expected feasibility (open-ended questions) were surveyed at baseline, and feasibility was assessed at follow-up (closed- and open-ended questions) and in the interviews. State well-being (affective and cognitive), state stress, state as well as retrospective time-use were measured in the EMA surveys. Compliance and feasibility were examined using a combination of quantitative (descriptive analyses) and qualitative methodologies.

**Results:**

Participants completed an average of 83% (SD 13%) of the daily surveys. Compliance varied by gender and age, where men (90% vs 80%) and older parents showed higher rates. Participants generally found the survey frequency and length manageable, though some suggested adjustments to the study period depending on their individual routines. The 7:30 AM survey was reported as the most challenging due to childcare drop-offs (40%-49%), followed by the 16:30 PM survey for similar reasons (7%-17%). The qualitative analysis further revealed additional points for improvement, for instance, the need for personalization (eg, individual adjustment of the survey timings and intervals), technical support, and the incorporation of gamification elements. Most interviewees (46% vs 23%) found the used measurement of well-being and stress to be appropriate. Regarding time use, they felt that the predefined activity groups (eg, personal care, working) were suitable (46%) but noted challenges assigning less frequent activities (eg, medical appointments) (5%-54%). Reporting the timings of time-use via consecutive questions (ie, specifying the duration or start and end times of an activity) was perceived as confusing (9%-69%), with participants expressing a preference for a visual overview, such as a Gantt chart.

**Conclusions:**

The study demonstrates that, when accounting for certain sociodemographic and study design factors, EMA can be a feasible method for data collection regarding daily well-being and time use, even in highly time-constrained populations like parents. This shows great potential for future research, such as exploring work-family conflict or performative gender roles and complementing established methods (eg, retrospective daily diaries).

**Trial Registration:**

OSF Registries osf.io/8qj3d; https://osf.io/8qj3d

**International Registered Report Identifier (IRRID):**

RR2-10.2196/54728

## Introduction

### Background

As parents often have to fulfill professional, domestic, and parental responsibilities, they can be considered a particularly burdened group [1]. These demands of balancing work, family, and personal life can lead to worse physical and mental health outcomes, underscoring the need for targeted support and interventions in daily life [2]. However, daily routines and associated (poor) well-being among parents have received relatively little attention, for instance, using real-time assessment methods, such as ecological momentary assessment (EMA) or experience sampling methods. Given that parents’ well-being is not only important for their own health but also for their children’s [3,4], further research into the feasibility and implementation of EMA in parents is important.

### Parents as a Target Group

Parents today face significant challenges by balancing childcare responsibilities, such as managing school drop-offs and pick-ups, with their careers. Although time use between men and women has become more similar in recent decades [5,6], substantial differences remain, with men generally spending more time on paid work and less on unpaid work [7-9]. As men continue to prioritize work, many women face a double burden, struggling to balance family and professional demands [7,9,10]. This discrepancy extends to care work and childcare. Women often reduce their paid work hours after giving birth, whereas men do not but may instead increase theirs [8]. Women’s share of housework is typically double that of men’s, with the largest gender differences observed among those with preschool-aged children [11,12]. Mothers experience more variation in how they use their time, often engaging in part-time work and childcare simultaneously, and activities frequently overlapping with others (eg, doing housework while taking care of the children) [13]. They generally also have less free time than fathers and tend to combine their leisure time with other activities, which can exacerbate stress and reduce opportunities for recovery [13].

Understanding how individuals behave in their everyday lives and how their actions affect mental and physical health is essential [2]. According to the Conservation of Resources Theory [14], individuals manage their time to preserve or enhance their resources (eg, energy, money, or social support), while the Work-Family Conflict Model [15] suggests that time is divided between work and family based on competing demands and conflicts and this conflict may lead to stress and poor health outcomes. The gender perspective complements these theories, examining how gender roles shape time use, with theories such as Doing Gender [16] and Gender Performativity [17] emphasizing the construction of gender roles through actions and behaviors. Previous research has established gender differences in daily activities and their impact on general health and well-being [7,9,10,18,19]. Certain activities—such as household cleaning and unpaid care work—are consistently related to worse health outcomes [20,21], and these are in turn more likely to be undertaken by women [9]. Therefore, examining the daily well-being and time use of parents, particularly mothers, is important for understanding and addressing gender disparities in health using a real-time lens.

### Ecological Momentary Assessment

The widespread use of smartphones provides an opportunity to collect real-time data and access a wider population. EMA involves “repeated collection of real-time data on participants’ momentary states in the natural environment” [19]. EMA can, for example, be implemented via smartphone apps, which can collect data both internet-based and offline and use various methods such as open-ended questions, text- or voice-based responses, Likert scales, multiple-choice options, number wheels, or sliders [22]. Data can be gathered at deliberately selected time points (ie, experience-based sampling, eg, after the morning coffee), randomly selected time points (ie, time-based sampling), or fixed intervals throughout the day (ie, interval-based sampling, eg, each morning at 9:00 AM).

While EMA has been widely used in clinical populations to monitor changes in state well-being during illness or substance withdrawal [23], its application among the population, for example, in parent samples, is limited. State well-being is relatively stable over time compared with mood [24], but there is a greater variation in positive and negative state well-being (eg, affect) than in cognitive state well-being (eg, life satisfaction) [25]. Overall, daily state well-being tends to decline throughout the day and toward the end of the week [26,27]. Understanding these patterns among parents and their association with daily routines and activities could inform interventions aimed at improving their well-being.

Although the research community has acknowledged the importance of daily activities for well-being and health, EMA is rarely implemented. So far, time use is generally measured through paper-pencil diary methods [28], studies that examine time-use patterns using EMA are rare [22,29]. EMA as opposed to paper-pencil diaries is better suited for capturing short and infrequent activities like errands and housework [29], which are precisely the areas where gender differences in time use are most pronounced, making EMA a valuable method for measuring these disparities. A comprehensive comparison of different methodological approaches is beyond the scope of this study, as we focus on the compliance and feasibility of the measurement of well-being and time use using EMA in a parent sample, without a control condition that uses daily diaries.

### Compliance and Feasibility of Ecological Momentary Assessment

Compliance is a key factor in the success of EMA studies, especially given the unpredictability of daily life. High compliance rates are essential for the validity of the data collected. Electronic EMA surveys have been shown to have higher compliance rates than paper-based methods (96% vs 70%), due to advantages such as automatic prompts, immediate data transfer, secure servers, and greater accessibility and convenience for participants [30]. Electronic assessment ensures that the responses are provided at the intended times, reducing issues with delayed or retrospective entries that can occur with paper-based methods. Higher compliance with electronic assessments has been found in both adults and children [31]. However, compliance rates can vary. De Vries and Bartels [32] reported an average compliance rate of 72%, ranging from 43% to 95%, while Passini et al [33] reported an average compliance rate of 83%. Factors such as the frequency of prompts and asking repeating questions can affect compliance; studies with higher prompt frequencies tend to have lower compliance rates [30]. There were no significant differences in compliance when using personal smartphones versus research-provided devices, nor did incentives influence compliance in app-based EMA applications [32].

### Compliance and Feasibility of Ecological Momentary Assessment in Parents

Parents, due to their high levels of stress and busy schedules, present unique challenges for EMA studies. Stress has been shown to impair episodic memory [34], suggesting that real-time data collection methods like EMA could be particularly advantageous for this group by reducing reliance on memory and recall.

Studies have shown that EMA is a feasible method for researching parenting behaviors. For instance, Passini et al [33] found that using EMA to record parenting behaviors 3 times a day was effective in capturing different dimensions of parenthood. In their study, 90% of mothers reported low difficulties using EMA, and 79% did not perceive it as burdensome. In addition, 60% would recommend EMA studies to other mothers. However, 74% indicated that the EMA study was at least partly challenging, suggesting a need to explore ways to reduce these challenges for parents.

Compliance rates in EMA studies involving parents vary. Kerr et al [35] reported that mothers responded to an average of 34 out of 50 prompts (ie, 68%) while El Dahr et al [36] reported a compliance rate of 82% in parents. Compliance was shown to be higher in the evenings [36,37].

Technical issues can also affect compliance; about 18% of EMA prompts are not answered due to problems such as data loss, battery difficulties, software bugs, or prompts occurring outside participants’ wake times [37]. Socioeconomic factors play a role as well, for example, Dzubur et al [37] found that mothers with higher annual incomes responded to prompts more quickly than those with lower incomes. This suggests that financial stability may afford parents more flexibility or resources to engage with EMA studies.

These findings suggest that while EMA is a promising method for studying parental well-being and time use, there are challenges that need to be addressed to enhance compliance and feasibility. Understanding the specific needs and constraints of parents is essential for designing EMA studies that are both effective and participant-friendly.

### Research Aims and Research Questions

The following research questions (RQ) were posed:

RQ1: What is the overall feasibility of parents participating in an EMA study measuring daily well-being and time use?

RQ2: What are the overall compliance rates for parents participating in an EMA study measuring daily well-being and time use?

RQ3: Which aspects of the study design (eg, time point) and sociodemographic factors are associated with noncompliance and feasibility?

## Methods

### Study Design

This work is part of a quasi-experimental pilot study using EMA [38]. The main study consisted of internet-based questionnaires (before and after a week of EMA), daily EMA surveys over the course of 7 days, and subsequent structured interviews for the intervention group. The control group only took part in the internet-based questionnaires and did not partake in the interviews or the EMA surveys. This study focuses on the intervention group; for the full study design see Altweck et al [38].

The minimum sample size (ie, 64 participants) for the intervention group was calculated using G*Power (version 3.1.9.6; Erdfelder, Faul, and Buchner; ANOVA, repeated measures, within a group, α=.05, power=0.8, correlation among repeated measures=0.5, and effect size [well-being]=0.25 [39]). For the interviews, to achieve data saturation [40,41], we aimed to recruit 6-17 participants.

The mixed methods approach facilitated the examination of compliance rates and their associations with various factors (eg, sociodemographic characteristics and study design features), while also providing insights into the feasibility of the study design through short, open-ended questionnaire responses and in-depth interview answers. First, participants in the intervention group filled in an internet-based questionnaire. On the next day, they started filling in the EMA surveys (each approximately 5-7 min), which were completed on participants’ personal Android smartphones using the app movisensXS (2024; movisens GmbH). The surveys took place 4 times per day (7:30 AM, 12 PM, 4:30 PM, 9 PM) with a buffer of 30 minutes over a period of 7 days. These time points were selected to also test feasibility in high-stress situations, for example, at 7:30 AM when parents get their children ready for nursery or school before work. The first 2 days were classed as a familiarization period and were not included in the analyses. After one week, participants filled in another internet-based questionnaire. The first participants to complete the daily EMA surveys were invited to take part in a structured interview to comprehensively understand the feasibility of the study design and to be able to make adjustments if necessary, which was not the case.

### Sample and Recruitment

Parents with at least one child under 18 years and living in the household were included. Only parents over the age of 18 years, without foster children, and without children with a chronic illness or disability were included, as these factors have been shown to further increase the burden of parenting [42]. Participants also needed access to an Android smartphone, in order to complete the daily surveys, for technical reasons.

Participants were recruited primarily from the northeast region of Germany using convenience sampling by distributing leaflets to relevant groups (eg, parent groups, nurseries, and schools) and digital channels (eg, the university’s website and social media). In an initial phone conversation, participants were informed about the study goals and methods, informed consent was collected, and, finally, the inclusion and exclusion criteria were checked. The first participants who completed the follow-up questionnaire were invited to participate in an interview.

### Procedure and Instruments

#### Baseline Questionnaires

Participants first filled in an internet-based questionnaire, which took approximately 20-30 minutes to complete.

The following sociodemographic variables were assessed: gender (woman, man), age (in years), education (International Standard Classification of Education-97 [43]; low and medium: level 0-4 [reference], high: level 5-6), employment status (full-time work, part-time work, not working), partnership status (in partnership, single, other), number of children under 14 years (numeric), number of children between 14 and 18 years (numeric), and family time (average h/d spent on family commitments [eg, childcare, family time, and housework] on both a regular workday and a free day). In addition, the expected feasibility of the study design was measured using the 4 open-ended questions (see Multimedia Appendix 1 for full item wording). For instance, participants were asked about the integration of EMA into their daily life and the survey’s length and time points.

#### Daily Ecological Momentary Assessment Surveys

On the next day, participants began with the daily EMA surveys. Participants started the EMA surveys either on Tuesday or Thursday. The daily EMA surveys included assessments of cognitive (life satisfaction item from the WHO-QoL [World Health Organization Quality of Life]-8) [44], positive (happy, relaxed), and negative (nervous, downcast) affective well-being [45], and stress (a global measure of perceived stress) [46]. Also, current activity and the sequence of activities since the last survey time point were queried (activity categories according to Tomczyk et al [9]; eg, working, housework, childcare). In the final surveys of the day, received and desired social support [3] as well as state mindfulness (Multidimensional State Mindfulness Questionnaire) [47] was assessed. Participants answered between 12 and 27 items per survey point, depending on (1) the variation in time use between time points (ie, the number of different activities reported) and (2) the survey time point, with more items presented in the evening. For details, see Altweck et al [38] and for the full EMA survey, see Multimedia Appendix 2.

#### Follow-Up Questionnaires

A total of 8 days after the baseline questionnaire, participants filled out another internet-based questionnaire.

The feasibility of the study design was surveyed with a mix of closed- and open-ended questions. The questions were inspired and adapted from the existing literature that examined the feasibility of EMA methods [22,48-50] (see Multimedia Appendix 1 for full item wording and scales). An example item included “In my opinion...I was able to accommodate the survey time point at 7:30am”, which was rated on a scale from 1 (strongly disagree) to 5 (strongly agree). Participants were able to explain their choices in an open textbox.

#### Interviews

Finally, a subgroup of the intervention group was invited to participate in an internet-based, structured interview that lasted 20-30 minutes. The interview guide was developed based on previous qualitative research on usability and feasibility evaluations [51,52] and was tailored to the current procedure, including focusing on the specific survey time points. The interviews focused on the feasibility of the EMA survey method and study design in greater detail. The interview followed the following structure: (1) general assessment of the study design, (2) time expenditure and integration into everyday life, (3) contents of the EMA surveys, (4) effects of the study, and (5) general and conclusion; see Multimedia Appendix 3 for the full interview guide.

### Ethical Considerations

The pilot study [38] was approved by the Ethics Committee of the University Medicine Greifswald in July 2022 (BB 113/23) and was conducted following the ethical standards set by the 1964 Declaration of Helsinki, its later amendments, or similar standards. Informed verbal and written consent was obtained from each participant, informing them about how their data would be used and stored, and that their participation was pseudonymized. They were also informed that they could withdraw from the study at any time without repercussions. Ethical guidelines and general data protection regulations were adhered to concerning data usage and storage. Participants in the intervention group were offered a monetary incentive of €35 (US $36.63) for the completion of the baseline and follow-up questionnaires as well as at least 50% of the daily surveys, an additional €15 (US $15.70) if they completed at least 80% of the daily surveys, and an additional €10 (US $10.47) if they took part in the interview.

#### Analytical Approach

##### Analysis Software

The statistical analyses were conducted using jamovi (version 3.5) [53], while the qualitative analyses were conducted using MAXQDA (version 24; VERBI Software GmbH) [54].

##### Data and Sample Description

For the sample description, we calculated mean values and SD for continuous variables and relative frequencies for categorical variables. Comparisons across gender were made using *t* tests, chi-square tests, or, where relevant, nonparametric equivalents (eg, Fisher exact test, Mann-Whitney *U* test, Welch *t* test).

##### Compliance

We analyzed compliance following guidelines by Liao et al [30] and Dao et al [55], reporting overall compliance (number of overall completed prompts), attrition (completed prompts by survey day and time point), and latency (time from prompt to survey completion and time to complete individual surveys). Associations of compliance with relevant sociodemographic factors were examined.

##### Feasibility

Closed questions regarding feasibility were analyzed using descriptive statistics (see the Data and Sample Description section). For the evaluation of the interviews and open-ended questions in the questionnaires, qualitative content analysis according to Kuckartz and Rädiker [56] was used. The qualitative data from the baseline questionnaires (ie, expected feasibility), follow-up questionnaires (feasibility), and interviews (feasibility) were coded and categorized separately.

The audio or video data from the interviews were transcribed for further analysis. The research team reflected on and noted their previous assumptions regarding the sample, research questions, and expected results. Summaries for each participant were compiled to gain an initial overview of the qualitative data. Text segments or sense units were coded and categorized either into existing categories or new ones. The first version of the main categories was created deductively based on the questionnaire and interview questions. Once coding began, an inductive approach was adopted, allowing the categories to be refined or extended with subcategories according to the data. In the first stage of the coding process, the qualitative data was once fully coded by a single person using the main deductive categories. Throughout this process, the research team noted overlapping categories, possible amendments (ie, new categories or redundant ones), and potential subcategories. Following Kuckartz and Rädiker [56], interrater agreement was achieved through discussions within the research team. In the second stage of the coding process, one researcher coded the data using the final categories twice, 2 weeks apart.

## Results

### Data and Sample Description

**Figure 1:**
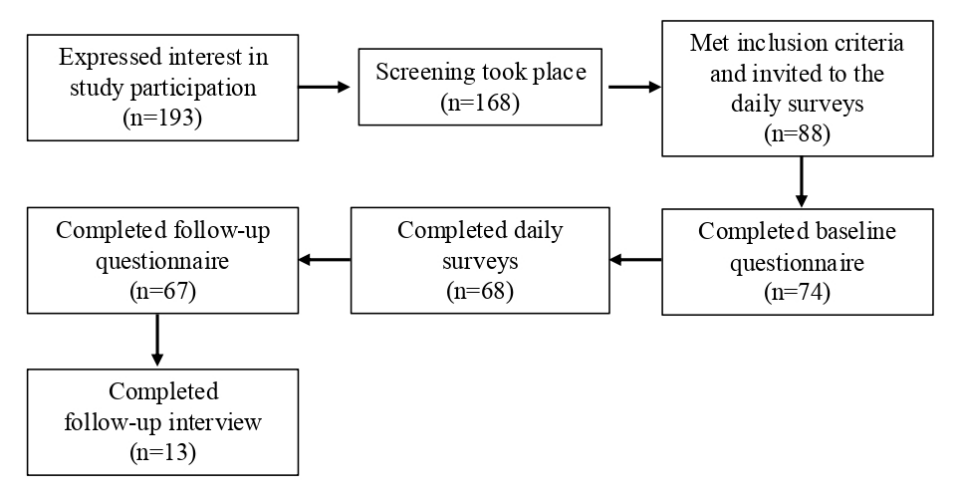
Sample overview including sample size and retention.

In total, 193 parents expressed interest in taking part in the study by leaving their personal information (Figure 1). We were able to reach 168 parents to complete the telephonic screening, of these, 88 met the inclusion criteria and were invited to the intervention group. In total, 74 parents completed the baseline questionnaires, 68 parents also completed the daily EMA surveys, 67 parents also completed the follow-up questionnaires, and 13 parents took part in the interviews. With 4 surveys per day over a 7-day period, we collected 1352 data points. To gain the most complete overview of the compliance and feasibility of this methodology in a parent sample, we included all possible cases in the analyses and excluded missing values case-wise (N=74). There was no missing data in the questionnaire and interview data, except average daily family time (2/74, 3%). The samples showed no significant differences in any sociodemographic variables or survey compliance (*P*>.05).

The final sample description is given in Table 1. The complete sample was made up of 77% (57/74) women and 23% (17/74) men. The average age was 37.6 (SD 5.9) years. The sample was highly educated (44/74, 60%), mostly in a relationship (53/74, 72%), and nearly equally divided across full-time, part-time, and no employment. On average, participants spent 9.6 (SD 4) hours per day on family commitments. There were few significant differences across genders, men were merely significantly older than women. Also, there were no significant differences across subsamples (baseline only, baseline and follow-up, interview group; *P*>.05).

**Table 1:** Descriptive statistics of the sample at baseline (total and by gender; N=74).

Sample variable				Total sample		Women		Men
**Age (years)**								
	Participants, n (%)		74 (100)		57 (77)		17 (23)	
	Mean (SD)		37.6 (5.9)		36.9 (5.1)		40.3 (7.6)^b^	
	Range		—^a^		28-53^b^		26-59^b^	
**Partnership status, n (%)**								
	In a relationship		53 (72)		39 (68)		14 (82)	
	Single		6 (8)		6 (11)		0 (0)	
	Other		15 (20)		12 (21)		3 (18)	
**Number of children**								
	Younger than 14 years							
		Mean (SD)	1.7 (0.8)		1.7 (0.8)		1.8 (0.8)	
		Range	1-4		1-4		1-3	
	Between 14 and 18 years							
		Mean (SD)	0.3 (0.5)		0.4 (0.6)		0.1 (0.4)	
		Range	0-2		0-2		0-1	
**Educational background, n (%)**								
	Low or medium		30 (41)		26 (46)		4 (24)	
	High		44 (60)		31 (54)		13 (77)	
**Employment status, n (%)**								
	Full-time		28 (38)		19 (34)		9 (53)	
	Part-time		29 (40)		24 (43)		5 (29)	
	None		16 (22)		13 (23)		3 (18)	
**Family time (h/d)**								
	Mean (SD)		9.6 (4)		10 (4.7)		8.2 (2.3)	
	Range		2-24		2-24		3-12.5	

^a^Not applicable.

^b^*P*<.05.

### Compliance

From the 28 maximum possible daily surveys to complete (4 times/d for 7 days), the average individual number of completed surveys was 23.1 (SD 3.57) (23.1/28, 82.5%; range 9-28) for results on compliance, refer to Table 2 and for grouped by time point or survey day, refer to Multimedia Appendix 4). Men (25/28, 90%; mean 25.1, SD 1.9) completed significantly more daily surveys than women (22.4/28, 80%; mean 2.4, SD 3.75) and the latter also showed greater variance (*t*_54.9_=3.87, *P*<.001). Neither the survey time point nor the survey day significantly affected compliance (*P*>.05).

**Table 2:** Descriptive statistics of compliance at follow-up (total and by gender; N=68).

Variable						Total sample						Women						Men			
						Mean (SD)			Range			Mean (SD)			Range			Mean (SD)			Range
Average completed surveys (app data)						23.1 (3.6)			9-28			22.4^a^ (3.8)			—^b^			25.1^a^ (1.9)			—
**Time to respond to trigger (app data)**																					
	**Across time points**				2.6 (17.4)			0-633			2.3 (8.9)			0-270			3.3 (30.9)			0-633	
		7:30 AM			2.6 (13.9)			0-270.2			3 (16.5)			0-270.2			1.6 (3.1)			0-22.2	
		12 PM			1.7^c^ (3.1)			0-26.1			1.7 (3.1)			0-26.1			1.5 (3.1)			0-21.7	
		4:30 PM			2.7^c^ (5.3)			0-34.7			3 (5.5)			0-28.5			2 (5)			0-34.7	
		9 PM			3.4 (31.6)			0-632.5			1.6 (3.5)			0-25.6			8 (61.3)			0-632.6	
**Time to complete the survey**																					
	Reported in the interviews				4.1 (1.8)			1.8-5.8			3.5 (2)			1.8-5.8			4.7 (1.5)			3.3-5.6	
	**App data**																				
		**Across time points**			3 (6.6)			0.4-137			3.2 (6.8)			0.5-137			2.8 (6.7)			0.4-134	
			7:30 AM	2.3^d^ (3.4)			0.4-60.2			2.4^e^ (4)			0.7-60.2			2.2^e,f^ (1.5)			0.4-11.3		
			12 PM	2.8^g^ (7.7)			0.4-134.4			2.8 (5)			0.6-60.4			3^h^ (13)			0.4-134.4		
			4:30 PM	2.4^i^ (3)			0.5-42.7			2.4^j^ (4.4)			0.5-42.7			2.2^j,k^ (1.8)			0.5-9.3		
			9 PM	4.7^d,g,i^ (9.6)			1.1-136.8			5.1^e,j^ (11.4)			1.2-136.8			3.8^e,f,h,j,k^ (2.7)			1.1-20.5		

^a^Significant differences across gender (*P*<.05).

^b^Not applicable.

^c^Significant differences across time points (*P*<.05).

^d^Significant differences across time points (*P*<.05).

^e^Significant differences across time points (*P*<.05).

^f^Significant differences across time points (*P*<.05).

^g^Significant differences across time points (*P*<.05).

^h^Significant differences across time points (*P*<.05).

^i^Significant differences across time points (*P*<.05).

^j^Significant differences across time points (*P*<.05).

^k^Significant differences across time points (*P*<.05).

Age significantly predicted the average individual number of completed surveys, where older participants showed higher compliance (*r*=0.33, *P*=.006). Other sociodemographic factors were not significantly related to compliance (*P*>.05).

On average, participants took 2.6 (SD 17.4) minutes to respond to a survey after receiving a signal; there was no significant gender difference (*P*>.05). Both men and women took longer to respond to the survey at 4:30 PM compared with the 12 PM one.

In the qualitative interviews, participants reported taking 4.1 (SD 1.8) minutes to complete the survey per time point, yet the app data showed that, on average, it took participants 3 (SD 6.6) minutes (see Multimedia Appendix 5 for examples and descriptions); there were no gender differences (*P*>.05). Both men and women took significantly longer to fill out the evening surveys (mean 4.7, SD 9.6) than the other time points (mean 2.3-2.8, SD 3.4-7.7); this discrepancy was also reported in the qualitative interviews.

### Feasibility

#### Technical Aspects

Interviewees found the app easy to use and user-friendly, which was confirmed in the follow-up questionnaires. Participants highlighted the reminder function of the surveys and appreciated the real-time support provided during the study by the research team. However, some technical issues were raised, including compatibility problems with the operating system, display issues, unreliable reminders, and the nonautomatic snooze feature. For more details regarding technical aspects see Multimedia Appendix 6.

#### Study Design

In terms of the overall feasibility of the study design, at baseline, 74% (53/74) did not report any expected concerns, and 13% (9/74) expressed excitement about taking part in the study questionnaires (see Multimedia Appendix 7 for qualitative results from the baseline questionnaires). However, most participants felt that the study design was generally “doable” (high feasibility: 20/74, 28%, moderate feasibility: 51/74, 71%) but expected certain difficulties in integrating the study into their daily lives (eg, some parts of the study design like a certain time point) and reported concerns, mainly around time management. The study duration (ie, the length of the overall study period and the individual surveys) appeared acceptable.

Participants found answering the questions every day adequately easy (mean 3.37, SD 1.04; scale: 1=difficult to 5=easy; refer to Tables 3 and 4 for quantitative results from the follow-up questionnaires). Some participants in the follow-up questionnaires (3/57, 5%) and 92% (12/13) interviewees reported a habituation effect because after a while “you already knew what you had to answer” (see Multimedia Appendix 8 for qualitative results from the follow-up questionnaires). Most interviewees reported the length and frequency of the individual survey time points to be adequate (12/13, 92% and 10/13, 77%, respectively). In the follow-up questionnaires, participants felt that the length of the individual time points was adequate, leaning slightly toward too much (mean 2.63, SD 0.8; scale: 1=too much to 5=far too little) but did not feel that the number of prompts was “annoying” (mean 2.48, SD 1.20; scale: 1=strongly disagree to 5=strongly agree). Most participants also did not feel that the study period was too long (mean 1.71, SD 0.94; scale: 1=strongly disagree to 5=strongly agree) and expressed that the survey period was adequate. However, 54% (7/13) of interviewees expressed a desire for the EMA study period to be longer than one week. Some expressed a desire for a longer survey period between 10 days and 3 weeks in order to better reflect fluctuations in everyday life and well-being. In contrast, one interviewee preferred a shorter survey period of 3 or 4 days, stating that the participation was stressful. In the follow-up questionnaires, 19% (11/57) of participants also requested a longer survey period, with the caveat that the number of daily surveys is reduced.

**Table 3:** Descriptive results from the follow-up questionnaires regarding feasibility (N=67).

Compliance		Mean (SD)
**I found answering the questions everyday...** (1=difficult to 5=easy)		3.37 (1.04)
**I found the length of the daily surveys to be...** (1=too much to 5=far too little)		2.63 (0.8)
**In my opinion...** (1=strongly disagree to 5=strongly agree)		
	...the duration of the test (7 d) was too long.	1.71 (0.94)
	...the number of daily prompts that were sent (4 times/d) was annoying.	2.48 (1.2)
	...I ignored or postponed the prompts.	1.75 (1.01)
	...the questions were understandable.	3.33 (1.04)
	...I was able to remember the activities since the last measurement time.	4.17 (0.94)

**Table 4:** Descriptive results from the follow-up questionnaires regarding the feasibility of the survey time points (N=67).

Feasibility		Total sample, mean (SD)	Men, mean (SD)	Women, mean (SD)
**In my opinion, I was able to accommodate the survey time at...well.** (1=strongly disagree to 5=strongly agree)				
	...7:30 AM...	3.42^a,b^ (1.33)	3.76 (1.39)	3.31 (1.29)
	...12 PM...	4.06^a,c^ (1.16)	4.18 (1.07)	4.02 (1.2)
	...4:30 PM...	3.62^c^ (1.24)	3.35 (1.32)	3.71 (1.21)
	...9:30 PM...	3.88^b^ (1.07)	4.24^d^ (0.66)	3.76^d^ (1.16)

^a^Significant differences across time points (*P*<.05).

^b^Significant differences across time points (*P*<.05).

^c^Significant differences across time points (*P*<.05).

^d^Significant differences across gender (*P*<.05).

#### Survey Time Points

In the baseline questionnaires, 8% (6/74) of participants anticipated that it would be difficult to integrate the survey time points in general into their daily life. About half of the interviewees (7/13, 54%) found it difficult to fill out the surveys at the predetermined time points. Most interviewees (9/13, 69%) found the 30-minute response time sufficient, while 15% (2/13) preferred a longer response window of one hour. In the follow-up questionnaires, participants generally reported that they did not ignore or postpone survey prompts (mean 1.75, SD 1.01; scale: 1=strongly disagree to 5=strongly agree).

In the baseline (29/74, 40%), follow-up questionnaires (28/57, 49%), and the interviews (6/13, 46%), the 7:30 AM time point was reported to be the single most difficult survey to complete “because most of the time it’s really the children’s drop-off time. So if you have school-age children (laughs) or have to take them to the nursery, it was always a bit difficult to fit that in somehow”. Instead, they suggested moving the early morning survey either a bit earlier (eg, 6:30 or 7:00 AM) or later (eg, 8:00 or 9:00 AM). The survey time point at 7:30 AM was said to be the hardest to accommodate, especially compared with 12 PM and 9:00 PM (*P*<.05).

At baseline, the 12 PM time point was expected to be feasible (15% vs moderate-low feasibility: 10%), but at follow-up, it showed mixed feasibility (15% vs moderate-low feasibility: 10%). It was feasible if it took place during the lunch break at work but bad if it coincided with work duties or the children’s lunchtime nap. For this time point, participants also suggested earlier (eg, 11:30 AM) or later times (eg, 2:00 PM).

In the baseline questionnaires, participants anticipated the time point at 4:30 PM to be the second most difficult to complete (17% vs moderate-low feasibility: 7%), which was confirmed at follow-up (high feasibility: 2%, low: 7%). The survey time point at 4:30 PM was also significantly easier to accommodate compared with 12 PM (*P*<.05). This time point was reported to be difficult if it coincided with the end of work and picking up the children from nursery or school or if it was after work and so the parents were in the middle of childcare and running errands or taking children to afternoon classes. Participants generally suggested a time a bit later, such as 5:00 PM or 5:30 PM.

In the interviews, the 9:00 PM time point was reported to be the second most feasible, whereas, in the follow-up questionnaires, 9:00 PM was reported to have been difficult to complete (high feasibility: 4% vs low feasibility: 19%). For some, this time point was “too late.” If the children were already asleep, this time point appeared to be the most feasible; however, if the parents were still getting the children ready for bed, as the children sleep late in general or just on that day, then this time point appears to be similarly difficult as the one at 7:30 AM. The only significant gender difference in the feasibility of time points was regarding 9:00 PM, which men found easier to accommodate than women (*P*<.05).

Participants mentioned a difference in feasibility between weekdays and weekends; for instance, the 7:30 AM time point was too early on weekends. They also expressed a desire for more flexibility in tailoring the survey points to individual routines.

#### Measurement of Well-Being and Time Use Using Ecological Momentary Assessment

Generally, participants reported that the questions in the daily surveys were adequately understandable (mean 3.33, SD 1.04; scale: 1=strongly disagree to 5=strongly agree).

Most interviewees (46% vs 23%) found the used measurement of well-being to be appropriate, describing the questions as “multifaceted,” while some reported the opposite and for example, felt that the items were too “superficial.” Instead, 23% (3/13) reported that the questions did not reflect fluctuations over the day or the week.

Most interviewees (10/13, 77%) agreed that the query about the current activity 4 times per day was not a full reflection of the full day and that the questions about the activities between assessments were necessary to complete the picture: “I think the combination really makes the most sense, because then you really get everything packed into it.”

There was some uncertainty regarding the predefined time-use categories. About half of the interviewees expressed uncertainty about assigning rare activities, for example, a doctor’s appointment, to a certain category, which was also mentioned in the follow-up questionnaires. Others were unsure how to classify leisure and physical activity, that is, whether to assign this to hygiene, eating, etc., or childcare, when, for example, watching TV while the children were around. Some participants also reported that they would have preferred logging the individual activities (eg, watching TV) rather than an activity group (eg, free or leisure time).

At baseline, 83% (60/74) expected to be able to remember the activities since the last survey time point, this was confirmed in the follow-up questionnaires (mean 4.17, SD 0.94; scale: 1=strongly disagree to 5=strongly agree) and interviews (12/13, 92%). Participants were asked to report what they were currently doing and then the sequence of what they had been doing since the last survey. Both in the interviews and the questionnaires, participants mentioned that this was confusing and they would have preferred reporting activities in chronological order. Also, some felt the sequential report of activities was redundant, for example, when they were at work at 12 PM and still at work at 4:30 PM. Participants were also able to click on multiple activities at the same time, while some found this useful, others found it confusing to report the sequence in this way. Participants also reported being unsure whether to report the time (eg, 7:05 AM) or the time passed in hours and minutes (eg, since 1 h 30 min); there was no clear preference. In both the follow-up questionnaire and interviews, participants suggested including a visual overview of the activity sequence, similar to a “Gantt chart.”

#### Miscellaneous

All interviewees also deemed the monetary incentive as appropriate; however, some also noted that as parents are short on time, a slightly higher incentive might be useful (between €50 [US $52.33] and €100 [US $104.65]). Some interviewees also mentioned that because the incentive was proportional to completed daily surveys, they would have liked a personal overview of their compliance.

One interviewee also mentioned that an offer of psychological support would be useful for participants who notice constant poor well-being or low levels of social support while completing the study.

Overall, the results offer a few key recommendations for future EMA studies with similar aims (Textbox 1). Ideally, these aspects should be discussed with the target group via participatory co-design workshops when preparing the study to ensure high commitment and compliance from the beginning.

**Key recommendations for designing ecological momentary assessments of time use and well-being in parents based on participants’ perspectives.**

- Technical support: Incorporate reminder functions and automatic snooze functions to facilitate completion and provide swift and adequate technical support throughout the study.
- Assessment schedule: Choose intervals that are acceptable for participants (eg, 4 times/d for 7 days or longer time periods with reduced frequency, eg, 2-3 times/d for 10-14 days).
- Customization: If possible: allow flexibility (eg, participants set their own time points within prespecified time windows).
- Visualization: Use visual aids, such as Gantt charts to illustrate sequential time use patterns instead of long (dropdown) lists’. Discuss whether to include open-ended or self-labelled responses and allow the logging of multiple overlapping activities.
- Incentives: Prepare for sufficient incentives as an appreciation for participants (eg, financial incentives of about €50 [US $52.33] per person).
- Gamification: If feasible, include elements of gamification (eg, live updates on compliance rates, positive feedback after completing a survey).

## Discussion

### Compliance and Feasibility

This study examined compliance and feasibility of an EMA of parental time use and well-being. By using qualitative (interviews, open-ended questions) and quantitative (EMA use data, surveys) methods, the study found that compliance was high (with an average of 83% [23.1/28] completed prompts) and feasibility was good for most parents that participated in the study. Older and male participants showed higher compliance. Participants also discussed several challenges to implementation regarding technical issues (eg, system compatibility and missing notifications), integration into daily life (eg, time points and frequency of assessments), and personal preferences (eg, how to log and classify daily activities).

The findings largely correspond to previous research that found similar compliance rates [30,33,36] and also reported a substantial amount of nonresponders due to technical issues [37]. However, in contrast to previous studies, we observed differences between fathers and mothers, with higher compliance rates and acceptance of evening assessments in fathers. Such differences were not found in other reviews that analyzed gender differences in EMA studies [57]. It is possible that mothers experienced excessive burden compared with fathers due to them more often having to balance family and professional demands [7,9,10] and having more informal care duties than fathers, which would give them less time to complete the assessments. Furthermore, fathers primarily having work duties would explain their frustration with repeated time use assessments during working hours (ie, monotonous responses at 12 PM and 4:30 PM), and would give them more time to complete evening assessments compared with mothers. However, this is merely speculative, and it is of interest to further examine time management and work-family conflict [15] in association with compliance and feasibility in the future. If work-family conflict affects well-being and time management, this could also be visible in daily life. Our sample was also highly educated, and previous research [37] found that mothers with higher annual incomes responded to prompts more quickly. This could indicate a bias and more research is needed in more diverse samples along different socioeconomic gradients.

Another important issue raised by participants referred to the customization of the assessment (eg, how to log and classify daily activities) and possible unintended consequences (eg, stress due to continued exposure to low well-being or visualization of work-family conflict in the time-use assessment). Since customization is in line with increased app engagement [58], this could be a good way to maintain compliance over time. However, this also requires more resources for the development, implementation, and data analysis. Therefore, more research is needed to explore the trade-off between complexity, compliance, and feasibility of the EMA design.

In addition, so far unintended consequences of such assessments have rarely been addressed in this target group, although research suggests that reactivity due to EMA monitoring is low [59,60]. Nevertheless, it is recommended to provide psychosocial support or resources in case of continuous reports of low levels of well-being. Since this can also affect compliance, additional incentives or reminders might be useful if multiple consecutive surveys are skipped or not completed. Other recommendations include extended training of participants to ensure a common understanding of data collection procedures. Fluctuations in well-being and negative affect can also influence app engagement. For example, parents may be less able to comply with EMA protocols on days when stress levels are higher, when their mood is lower or their children’s behavior is more difficult to manage. There is also a possibility of potential reporting bias due to measurement reactivity as well as the risk that parents might skip surveys.

This has implications for ecological momentary interventions and just-in-time adaptive interventions: For instance, participants experiencing high levels of stress might benefit from a brief stress management intervention (eg, breathing techniques and mindfulness minute) to support their momentary emotion regulation before completing a more complex survey. This could not only improve their well-being but also their compliance. On the other hand, participants reporting positive mood might benefit from further reinforcement (in the sense of an upward spiral of positive emotions, following the broaden-and-build theory [61]) to sustain their positive mood and also their compliance. Therefore, analyzing stress, well-being, and app compliance concurrently is useful to account for days with higher or lower levels of engagement and well-being. Future studies may also want to use different types of operationalization of well-being or stress [31] to identify key moments to increase engagement and ensure compliance. For instance, participants with low compliance could receive compliance-facilitating interventions, such as higher incentives or gamification elements, which could then lead to self-efficacy and a positive mood. In summary, we argue that looking more closely at the association between compliance, well-being, and stress might be beneficial to study quality (ie, lower attrition, higher retention rates) as well as participant well-being (eg, via EMIs or just-in-time adaptive interventions).

Finally, we present key recommendations for EMA designs assessing time use and well-being in parents (Textbox 1). These include ensuring robust technical support, tailoring assessment schedules to participants’ needs, and offering flexibility in survey timing. We also highlight the importance of effective visualizations, sufficient incentives, and incorporating gamification elements to enhance engagement and usability.

### Strengths and Limitations

The study is based on a convenience sample of parents, and therefore, it was not representative of the population. Particularly the female-dominant sample limits the generalizability of findings to fathers. In particular, parents with increased vulnerability (eg, few socioeconomic resources or children with a medical condition) were not part of the study, which could have biased the results. Furthermore, the assessment was limited to a few key questions for each outcome, which limits the scope of the examined constructs (eg, well-being). Furthermore, we did not compare different variations of the study design (eg, more or less frequent assessments, active vs passive data collection) and cannot generalize our findings to other methods or assessment schedules. Compliance and feasibility may differ between interval-contingent and random-signal-contingent sampling regimes, as parents in the former can mentally prepare and plan accordingly. Using brief scales and single-item measures is common and recommended in EMA research to limit the potential burden on participants, avoid dropouts, and achieve valid results [32]. Since we assumed parents to be a high-burdened group, because of demands from both family and work environments, it was important to us to use a brief survey for each EMA prompt. We also used psychometrically validated and previously tested items whenever possible to increase the comparability and methodological quality of the assessment. To explore other potential weaknesses and strengths of the study design, we also asked open-ended questions and conducted qualitative interviews to explore participants’ perspectives and gain a better understanding of the challenges they face in this type of study.

### Conclusion

In summary, our pilot study demonstrates that EMA methodologies are valuable for investigating parental time use and well-being in everyday life. They present unique opportunities for future research, such as exploring work-family conflict or performative gender roles, complementing established methods like daily diaries. In addition, they enable advanced big data analyses (eg, sequential cross-lagged panel models, sequence-based grouping algorithms) and support data-driven interventions that address the complex relationship between voluntary and involuntary daily activities, gender, and health.

## Appendix

Multimedia Appendix 1 Questions regarding feasibility in the baseline and follow-up questionnaires.

Multimedia Appendix 2 Full ecological momentary assessment questionnaire.

Multimedia Appendix 3 Full interview guide.

Multimedia Appendix 4 Compliance by survey day and time point.

Multimedia Appendix 5 Frequency and description of the themes from the interviews (N=13).

Multimedia Appendix 6 Technical aspects.

Multimedia Appendix 7 Frequency and description of the themes from the baseline questionnaires (N=74).

Multimedia Appendix 8 Frequency and description of the themes from the follow-up questionnaires (N=57).

## Abbreviations

EMA: ecological momentary assessment
WHO-QoL: World Health Organization Quality of Life

## Data Availability

The datasets generated or analyzed during this study are available from the corresponding author on reasonable request.
