# Supplementary material for: Ecological Momentary Assessment of Parental Well-Being and Time Use: Mixed Methods Compliance and Feasibility Study
Source: JMIR Form Res. 2025 Apr 23;9:e67451. doi: 10.2196/67451 (PMC12059499; doi:10.2196/67451)
Supplement: Multimedia Appendix 1 [file formative_v9i1e67451_app1.docx]

**Multimedia appendix 1.** Questions regarding feasibility in the baseline and follow-up questionnaires

**Erwartete Machbarkeit (Baseline Fragebogen)**

Sie werden über die nächsten 7 Tage viermal täglich befragt (7:30 Uhr, 12:00 Uhr, 16:30 Uhr und 21:00 Uhr plus einen Toleranzrahmen von 30 Minuten).
An den jeweiligen Erhebungszeitpunkten werden Sie zu Ihren gegenwärtigen Aktivitäten und Befinden befragt. Die tägliche Abfrage dauert ca. 5 Minuten und abends ca. 8 Minuten.
Zunächst möchten wir Ihre Gedanken, Erwartungen und ggf. Bedenken zu den täglichen Befragungen erfahren.

Ihrer Meinung nach, inwiefern lassen sich die täglichen Befragungen in Ihren Alltag integrieren?
________________________________________

Was denken Sie zu Umfang und Zeitpunkten der täglichen Befragungen?

________________________________________

Was denken Sie, wie gut Sie sich an die Aktivitäten in der vorherigen Erhebungszeit erinnern können?

________________________________________

Haben Sie weitere Gedanken oder Bedenken zum Ablauf?
_______________________________________

**Machbarkeit (Follow-up Fragebogen)**

Runyan, J. D. et al. (2013). A smartphone ecological momentary assessment/intervenNon “app” for collecting real-time data and promoting self-awareness. PloS one, 8(8), e71325.

Die Fragen täglich zu beantworten fiel mir....
Skala von 1 (schwer) bis 5 (leicht)

Den Umfang der täglichen Abfrage empfand ich als:
1 zu viel
2 etwas zu viel
3 genau richtig
4 etwas zu wenig
5 viel zu wenig

Auf einer Skala von 1-10, wie gut hat die mobile Anwendung/App (movisensXS) funktioniert?
Auf einer Skala von 1-10, wie intuitiv war die Nutzung der mobilen Anwendung/App
(movisensXS)?
SKALA: 1 schlecht - 10 gut

Messner, E. M., Terhorst, Y., Barke, A., Baumeister, H., Stoyanov, S., Hides, L., ... & Probst, T. (2020). The German version of the Mobile App RaNng Scale (MARS-G): development and validaNon study. JMIR mHealth and uHealth, 8(3), e14479.

Wie leicht ist es den Umgang mit der mobilen Anwendung/App (movisensXS) zu erlernen?
1. Keine oder unzureichende Erklärungen; Menübeschreibungen und Symbole sind verwirrend und/
oder kompliziert.
2. Nach hohem Aufwand benutzbar.
3. Nach einigem Aufwand benutzbar.
4. Benutzung ist leicht erlernbar oder movisensXS weist klare Instruktionen zur Benutzung auf.
5. Eine Intuitive und einfache Benutzung movisensXS ist sofort möglich.

Spook, J. E., Paulussen, T., Kok, G., & Van Empelen, P. (2013). Monitoring dietary intake and physical activity electronically: feasibility, usability, and ecological validity of a mobile-based Ecological Momentary Assessment tool. Journal of medical Internet research, 15(9), e214.

Meiner Meinung nach ...
1. war es langweilig, mit der mobilen Anwendung/App (movisensXS) zu arbeiten.
2. war die mobile Anwendung/App (movisensXS) einfach zu bedienen.
3. waren die Fragen verständlich.
4. habe ich die Aufforderungen ignoriert oder aufgeschoben.
5. konnte ich mich an die Aktivitäten seit dem letzten Messzeitpunkt gut erinnern.
6. dauerte der Versuchsdauer (7 Tage) zu lange.
7. war die Anzahl der Aufforderungen, die während einer Woche (viermal am Tag) gesendet wurden,
lästig.
8. konnte ich den Messzeitpunkt um 7:30Uhr gut einrichten.
9. konnte ich den Messzeitpunkt um 12:00Uhr gut einrichten.
10.konnte ich den Messzeitpunkt um 16:30Uhr gut einrichten.
11.konnte ich den Messzeitpunkt um 21:00Uhr gut einrichten.

Skala von 1 (stimme überhaupt nicht zu) bis 5 (stimme voll und ganz zu)

Meiner Meinung nach konnte ich den Messzeitpunkt um…

1. 7:30 Uhr gut einrichten.
2. 12:00 Uhr gut einrichten.
3. 16:30 Uhr gut einrichten.
4. 21 Uhr gut einrichten.

Skala von 1 (stimme überhaupt nicht zu) bis 5 (stimme voll und ganz zu)

Wenn Item 1), 4), 6), 7, 8), 9), 10), 11) aus der vorherigen Aufgabe: Stimme eher zu/Stimme voll und ganz zu (4/5)

*Warum?*________________________________________________

Wenn Item 2), 3), 5) aus der vorherigen Frage: Stimme überhaupt nicht zu/Stimme eher nicht zu (1/2)

*Warum nicht?*__________________________________________________

Ich würde folgende Messzeitpunkte ändern, weil...
_____________________

Ich würde folgende Versuchsdauer planen...
_____________________

Ich würde folgende Änderung an der mobilen Anwendung/App (movisensXS) vornehmen...
_____________________

Ich würde folgendes zur mobilen Anwendung/App (movisensXS) hinzufügen...

_____________________
