# Supplementary material for: Ecological Momentary Assessment of Parental Well-Being and Time Use: Mixed Methods Compliance and Feasibility Study
Source: JMIR Form Res. 2025 Apr 23;9:e67451. doi: 10.2196/67451 (PMC12059499; doi:10.2196/67451)
Supplement: Multimedia Appendix 2 [file formative_v9i1e67451_app2.docx]

**Multimedia appendix 2.** Full EMA questionnaire

**Erhebungsinstrumente**

**EMA-Befragung (T1-T7, nur Interventionsgruppe)**

**Aktuelles Wohlbefinden**

Blanke, E. S., Riediger, M., & Brose, A. (2018). Pathways to happiness are multidirectional: Associations between state mindfulness and everyday affective experience. *Emotion*, *18*(2), 202.

- Bitte bewerten Sie, wie gut die folgenden Adjektive Ihre Gefühle zum aktuellen Zeitpunkt beschreiben.
  - glücklich
  - entspannt
  - nervös
  - deprimiert

Skala 0 (trifft überhaupt nicht zu) bis 6 (trifft stark).

Item WhoQol-8

- Wie zufrieden sind Sie aktuell mit Ihrem Leben?

Skala von 1= sehr unzufrieden bis 5= sehr zufrieden

**Stress**

Cohen, S., Kamarck, T., & Mermelstein, R. (1983). A global measure of perceived stress. Journal of Health and Social Behavior, 24, 385-396. <https://doi.org/10.2307/213640>

Die nachfolgenden Fragen beziehen sich auf Ihre Gedanken und Gefühle im aktuellen Moment.

| Wie sehr haben Sie aktuell das Gefühl, … |
| --- |
| wichtige Dinge in Ihrem Leben nicht beeinflussen zu können? |
| sicher im Umgang mit persönlichen Aufgaben und Problemen zu sein? |
| dass sich die Dinge nach Ihren Vorstellungen entwickeln? |
| dass sich die Probleme so aufgestaut haben, dass Sie diese nicht mehr bewältigen können? |

Antwortkala: 0 = gar nicht BIS 4 = sehr

**Zeitnutzung**

Tomczyk, S., Altweck, L., & Schmidt, S. (2021). How is the way we spend our time related to psychological wellbeing? A cross-sectional analysis of time-use patterns in the general population and their associations with wellbeing and life satisfaction. *BMC public health*, *21*, 1-9.

*(Seite 1)*

Uns interessiert a) was Sie aktuell machen und b) was Sie zwischen der letzten Befragung (d.h. 7:30Uhr / 12Uhr / 16:30Uhr / 21Uhr) und jetzt gemacht haben.

Die Abfrage erfolgt nacheinander.

*(Seite 2)*

Welche Aktivität machen Sie gerade in diesem Moment?
*Bitte zählen Sie den Weg zur Aktivität dazu (z. B. Arbeitsweg —> Arbeit)*(Mehrfachauswahl möglich, nur falls Sie mehrere Aktivitäten gleichzeitig machen)

- Arbeit
- Hausarbeit/ Besorgungen/ Reparaturen
- Kinderbetreuung und Organisation
- Unbezahlte Pflege Erwachsener (z. B. Großeltern)
- Studium/ Bildung
- Freizeit/ Körperliche Aktivität/ Hobbies
- Selbstfürsorge (z. B. Essen, Schlafen, Hygiene)

Seit wann machen Sie diese Aktivität? (Uhrzeit)

- - __ : __

*(Seite 3)*

Was haben Sie im Anschluss an die letzte Befragung gemacht? (d.h. 7:30Uhr / 12Uhr / 16:30Uhr / 21Uhr).

ENTWEDER:

1. Was ich jetzt mache (schon angegeben)

ODER: Wenn Sie etwas anderes gemacht haben, wählen Sie bitte die Aktivität aus, sonst lassen Sie es frei.
Bitte zählen Sie den Weg zur Aktivität dazu (z. B. Arbeitsweg —> Arbeit).

(Mehrfachauswahl möglich, nur falls Sie mehrere Aktivitäten gleichzeitig machen)

1. Arbeit
2. Hausarbeit/ Besorgungen/ Reparaturen
3. Kinderbetreuung und Organisation
4. Unbezahlte Pflege Erwachsener (z. B. Großeltern)
5. Studium/ Bildung
6. Freizeit/ Körperliche Aktivität/ Hobbies
7. Selbstfürsorge (z. B. Essen, Schlafen, Hygiene)

Seit wann machen Sie diese Aktivität? (Uhrzeit)

- - __ : __

*(Seite 4)*

Was haben Sie danach gemacht?

ENTWEDER:

1. Was ich jetzt mache (schon angegeben)

*(Seite 5)*

ODER: Wenn Sie etwas anderes gemacht haben, wählen Sie bitte die Aktivität aus.
Bitte zählen Sie den Weg zur Aktivität dazu (z. B. Arbeitsweg —> Arbeit).

(Mehrfachauswahl möglich, nur falls Sie mehrere Aktivitäten gleichzeitig machen)

- Arbeit
- Hausarbeit/ Besorgungen/ Reparaturen
- Kinderbetreuung und Organisation
- Unbezahlte Pflege Erwachsener (z. B. Großeltern)
- Studium/ Bildung
- Freizeit/ Körperliche Aktivität/ Hobbies
- Selbstfürsorge (z. B. Essen, Schlafen, Hygiene)

Seit wann machen Sie diese Aktivität? (Uhrzeit)

- - __ : __

*(Seite 6)*

Was haben Sie danach gemacht?

ENTWEDER:

1. Was ich jetzt mache (schon angegeben)

ODER: Wenn Sie etwas anderes gemacht haben, wählen Sie bitte die Aktivität aus.
Bitte zählen Sie den Weg zur Aktivität dazu (z. B. Arbeitsweg —> Arbeit).

(Mehrfachauswahl möglich, nur falls Sie mehrere Aktivitäten gleichzeitig machen)

- Arbeit
- Hausarbeit/ Besorgungen/ Reparaturen
- Kinderbetreuung und Organisation
- Unbezahlte Pflege Erwachsener (z. B. Großeltern)
- Studium/ Bildung
- Freizeit/ Körperliche Aktivität/ Hobbies
- Selbstfürsorge (z. B. Essen, Schlafen, Hygiene)

Seit wann machen Sie diese Aktivität? (Uhrzeit)

- - __ : __

**Art des Tages**

European Union (2019). Harmonised European Time Use Surveys (HETUS) 2018 Guidelines (2019 Ed.). Publications Office of the European Union. https://doi.org/10.2785/926903

- War heute ein ganz normaler oder ein ungewöhnlicher Tag ?

Gewöhnlicher Arbeitstag

Freier Tag durch Wochenende/Arbeitsplan

Krankheitstag

Urlaubstag

Beurlaubt aus anderen Gründen

**Tägliche Soziale Unterstützung**

Adaptaptiert aus:

Bischoff, M., Howland, V., Klinger-König, J., Tomczyk, S., Schmidt, S., Zygmunt, M., ... & Grabe, H. J. (2019). Save the children by treating their mothers (PriVileG-M-study)-study protocol: a sequentially randomized controlled trial of individualized psychotherapy and telemedicine to reduce mental stress in pregnant women and young mothers and to improve Child’s health. *BMC psychiatry*, *19*(1), 1-15.

Haben Sie heute soziale Unterstützung von Ihrem/r Partner/in erhalten?

"ja" "nein"

WENN JA:

Wie zufrieden sind Sie mit der Unterstützung, die Sie heute von Ihrem/r Partner/in erhalten haben?

SCALE " 1 2 3 4 5" "überhaupt nicht zufrieden" "sehr zufrieden”

Haben Sie sich heute mehr soziale Unterstützung von Ihrem/r Partner/in gewünscht?

SCALE " 1 2 3 4 5" "überhaupt nicht" "sehr"

Haben Sie heute soziale Unterstützung von wichtigen Bezugspersonen (z. B. Familie, Freunde) erhalten?

"ja" "nein"

WENN JA:

Wie zufrieden sind Sie mit der Unterstützung, die Sie heute von wichtigen Bezugspersonen erhalten haben?

SCALE " 1 2 3 4 5" "überhaupt nicht zufrieden" "sehr zufrieden”

Haben Sie sich heute mehr soziale Unterstützung von wichtigen Bezugspersonen gewünscht?

SCALE " 1 2 3 4 5" "überhaupt nicht" "sehr"

**State Achtsamkeit**

Michalak J, Zarbock G, Drews M, Otto D, Mertens D, Ströhle G, et al. Erfassung von achtsamkeit mit der deutschen version des Five Facet Mindfulness Questionnaires (FFMQ-D). *Z Gesundheitspsychol*. 2016;24(1):1-12.

Bitte denken Sie an den heutigen Tag: Wie haben Sie sich verhalten, was ging in Ihnen vor? Darauf beziehen sich die folgenden Fragen. (Awa)

Skala: 0 (trifft überhaupt nicht zu) – 6 (trifft sehr gut zu)

1. Ich habe Aufgaben/Dinge automatisch erledigt, ohne wirklich mitzubekommen, was ich tue.
2. Ich habe Dinge getan, ohne auf sie zu achten.
3. Ich habe manchmal nicht auf das geachtet, was im jeweiligen Moment passiert ist.
4. Ich habe gedacht, dass manche meiner Gedanken/ Gefühle etwas unpassend waren.
5. Mir sind Dinge durch den Kopf gegangen, die mich eigentlich nicht beschäftigen sollten.
6. Ich habe gedacht, dass ich mich in einem bestimmten Moment hätte besser verhalten können.
7. Ich habe meine Aufmerksamkeit auf den jeweils aktuellen Moment gerichtet.
8. Ich habe mich jeweils auf das eingelassen, was gerade geschah.
9. Ich habe mich auf das konzentriert, was ich gerade getan habe.
