# Supplementary material for: Ecological Momentary Assessment of Parental Well-Being and Time Use: Mixed Methods Compliance and Feasibility Study
Source: JMIR Form Res. 2025 Apr 23;9:e67451. doi: 10.2196/67451 (PMC12059499; doi:10.2196/67451)
Supplement: Multimedia Appendix 3 [file formative_v9i1e67451_app3.docx]

**Multimedia appendix 3.** Full interview guide

Herzlich willkommen und vielen Dank, dass Sie sich heute für das Interview die Zeit genommen haben. Sie haben bereits an der Studie “Tägliche Zeitnutzungsmuster und Lebensqualität von Elternt” teilgenommen, auch dafür möchten wir uns noch einmal bedanken. Heute würden wir gerne an Ihren Erfahrungen mit der Studie teilhaben. Ziel ist es, die Gestaltung weiter zu verbessern, sodass wir bei der Umsetzung in einer größeren Gruppe Problemen voraus kommen. Deswegen ist es wichtig für uns, dass Sie heute frei erzählen, was ihre Eindrücke von der Studie waren, egal ob diese besonders negativ, besonders positiv oder neutral waren. Alles was Sie heute sagen, bleibt vertraulich, Protokolle und Mitschriften werden pseudonymisiert angefertigt, sodass kein Rückschluss auf Ihre Person möglich ist.

Das Gespräch ist in verschiedene Themenblöcke unterteilt und wird ca. 30-45 Minuten in Anspruch nehmen.

Haben Sie Fragen zum Ablauf oder Anmerkungen vorab?

Prima! Dann werde ich jetzt die Aufnahme beginnen. *TON-/VIDEOAUFNAHME STARTEN*

Für die Befragungen haben Sie ein Pseudonym erstellt. Können Sie mir bitte ihr Pseudonym nennen?

*FALLS SIE SICH NICHT ERINERRN:* Als Erinnerung, das Pseudonym sollte anhand folgender Kriterien erstellt werden:

- die BEIDEN ERSTEN Buchstaben des Vornamens
- die BEIDEN ERSTEN Buchstaben des ersten Nachnamens
- die LETZTEN DREI Ziffern Ihrer Handynummer an

Beispiel: Lisa Hoffmann, 6775599732 —> FEHO732

**Generelle Einschätzung des Studiendesigns**

Zunächst möchte ich Ihnen ein paar Fragen zu Ihrer generellen Einschätzung des Studiendesigns stellen

Wie gut oder schlecht hat die mobile Anwendung/App (movisensXS) funktioniert?

Wie intuitiv war die Nutzung der mobilen Anwendung/App (movisensXS)?

Konnten Sie einen Lerneffekt feststellen und nach wiederholter Nutzung der App diese schneller bedienen?

Was fanden Sie besonders gut an der mobilen Anwendung/App (movisensXS)?

Was fanden Sie besonders schlecht an der mobilen Anwendung/App (movisensXS)?

Haben Sie sonst noch Anmerkungen zur Handhabung der mobilen Anwendung/App (movisensXS)?

**Zeitaufwand & Integration in den Alltag**

Als nächstes interessiert uns Ihre Meinung zum Zeitaufwand der Studie & deren Integration in den Alltag

Wie viel Zeit haben Sie jeden Tag mit dem Ausfüllen aller Fragebögen verbracht?

Halten Sie diese Zeit für angemessen?

Kamen die Abfragen in, für Sie, passenden Momenten, oder hätten Sie zu einem anderen Zeitpunkt stattfinden sollen?

—> Wenn es unpassend war, welche Zeitpunkte wären besser gewesen?

Wie empfanden Sie den 30-minütigen Zeitpuffer der einzelnen Befragungen? War dieser ausreichend?

Wie fanden Sie die Anzahl an Abfragen pro Tag?

War die Länge der Studie (d.h. täglich für 1 Woche) angemessen? Warum?

Haben Sie sonst noch Anmerkungen zum Zeitaufwand und Integration in den Alltag?

**Inhalte der Befragung**

Als nächstes möchte ich Ihnen ein paar Fragen zu den Inhalten de täglichen Befragungen stellen.

Waren die Fragen verständlich formuliert? Bitte erläutern Sie.

Gab es Aktivitäten, die Sie der Auswahl nicht zuordnen konnten oder bei derer Zuteilung Sie sich unsicher waren? Wenn ja, welche?

Hatten Sie das Gefühl, dass ihr Wohlbefinden bzw. Stressempfinden über den Tag hinweg adäquat gemessen wurde? Bitte erläutern Sie.

Konnten Sie sich gut an ihre vergangen Aktivitäten erinnern oder hatten Sie das Gefühl welche zu vergessen? Bitte erläutern Sie.

Ihrer Meinung nach, war die Momentabfrage der Aktivitäten repräsentativ für Ihren durchschnittlichen Alltag?Bitte erläutern Sie.

Haben Sie sonst noch Anmerkungen zum Inhalt der täglichen Befragungen?

**Auswirkungen der täglichen Befragungen**

Als nächstes möchte ich mit Ihnen über die potentiellen Auswirkungen, der täglichen Befragungen, auf Ihren Alltag sprechen.

Inwiefern, wenn überhaupt, gab es eine Veränderung in Ihrem eigenen Denken seit Beginn der Studie? Bitte erläutern Sie.

Inwiefern, wenn überhaupt, gab es eine Veränderung in Ihrem eigenen Verhalten seit Beginn der Studie? Bitte erläutern Sie.

Gab es eine Veränderung in Ihrem Wohlbefinden und/oder Stressempfinden? Bitte erläutern Sie.

Gab es eine Veränderung in sozialen Interaktionen mit Ihrer Familie und/oder Partner? Bitte erläutern Sie.

Haben Sie sonst noch Anmerkungen zu den potentiellen Auswirkungen der täglichen Befragungen?

**Allgemein**

Zuletzt möchten wir Ihr allgemeines Feedback zur Studie einholen.

Hatten Sie Bedenken beim Benutzen der mobile Anwendung/App (movisensXS) (z.B. im Bezug auf Datenschutz) ? Welche?

Auf welchem Weg haben Sie über diese Studie erfahren?

Wo würden Sie potentielle Studienteilnehmer*innen (d.h. Eltern) für eine solche Studie finden, wenn Sie diese selbst rekrutieren würden?

Sie können ja XXX...Wie angemessen fanden Sie die Aufwandsentschädigung?

Stellen Sie sich vor Sie sind Designer einer App, mit der Sie genau diese Studie durchführen wollen. Wie würden Sie diese (- nach ihrer jetzigen Testung der movisensXS-App -) besonders ansprechend und einfach zu bedienen gestalten?

Gibt es noch irgendwelche Anmerkungen, Ideen oder Gedanken, die Sie uns mitteilen wollen?

**Abschluss**

Das beendet den inhaltlichen Teil des Interviews. Ich werde die Aufnahme jetzt beenden. *TON-/VIDEOAUFNAHME BEENDEN*

Vielen Dank, dass Sie sich heute die Zeit genommen haben, um an diesem Interview teilzunehmen und ihre Gedanken mit uns zu teilen. Wir sind jetzt mit der Gespräch fertig, falls es etwas gibt, dass Ihnen später, im Bezug auf die Studie, noch einfällt, können Sie sich gerne an die Studienleitung via Mail wenden.

Einen schönen Tag noch!
