# Supplementary material for: Ecological Momentary Assessment of Parental Well-Being and Time Use: Mixed Methods Compliance and Feasibility Study
Source: JMIR Form Res. 2025 Apr 23;9:e67451. doi: 10.2196/67451 (PMC12059499; doi:10.2196/67451)
Supplement: Multimedia Appendix 4 [file formative_v9i1e67451_app4.docx]

**Multimedia appendix 4.** Compliance by survey day and time point

**Table 1.** Compliance grouped by survey day

| **Survey day** | **Gender** | **Missing** | | **Completed** | |
| --- | --- | --- | --- | --- | --- |
|  |  | ***n*** | **%** | ***n*** | **%** |
| 2 | Men | 5 | 7.35 | 63 | 92.65 |
|  | Women | 26 | 12.87 | 176 | 87.13 |
|  | Total | 36 | 12.59 | 250 | 87.41 |
| 3 | Men | 6 | 8.82 | 62 | 91.18 |
|  | Women | 25 | 12.25 | 179 | 87.75 |
|  | Total | 38 | 13.19 | 250 | 86.81 |
| 4 | Men | 7 | 10.45 | 60 | 89.55 |
|  | Women | 40 | 19.70 | 163 | 80.30 |
|  | Total | 52 | 18.18 | 234 | 81.82 |
| 5 | Men | 6 | 9.09 | 60 | 90.91 |
|  | Women | 35 | 17.41 | 166 | 82.59 |
|  | Total | 44 | 15.66 | 237 | 84.34 |
| 6 | Men | 7 | 10.45 | 60 | 89.55 |
|  | Women | 36 | 18.65 | 157 | 81.35 |
|  | Total | 49 | 18.01 | 223 | 81.99 |
| 7 | Men | 5 | 8.77 | 52 | 91.23 |
|  | Women | 18 | 13.53 | 115 | 86.47 |
|  | Total | 24 | 11.94 | 177 | 88.06 |

**Table 2.** Compliance grouped by time point

| **Time point** | **Gender** | **Missing** | | **Completed** | |
| --- | --- | --- | --- | --- | --- |
|  |  | ***n*** | **%** | ***n*** | **%** |
| 07:30 | Men | 6 | 6.12 | 92 | 93.88 |
|  | Women | 49 | 17.38 | 233 | 82.62 |
|  | Total | 62 | 15.46 | 339 | 84.54 |
| 12:00 | Men | 13 | 13.00 | 87 | 87.00 |
|  | Women | 45 | 15.46 | 246 | 84.54 |
|  | Total | 64 | 15.53 | 348 | 84.47 |
| 16:30 | Men | 9 | 9.18 | 89 | 90.82 |
|  | Women | 59 | 14.64 | 344 | 85.36 |
|  | Total | 41 | 14.44 | 243 | 85.56 |
| 21:00 | Men | 8 | 8.25 | 89 | 91.75 |
|  | Women | 45 | 16.13 | 234 | 83.87 |
|  | Total | 58 | 14,57 | 340 | 85.43 |
