# Supplementary material for: Ecological Momentary Assessment of Parental Well-Being and Time Use: Mixed Methods Compliance and Feasibility Study
Source: JMIR Form Res. 2025 Apr 23;9:e67451. doi: 10.2196/67451 (PMC12059499; doi:10.2196/67451)
Supplement: Multimedia Appendix 5 [file formative_v9i1e67451_app5.docx]

**Multimedia appendix 5.** Frequency and description of the themes from the interviews (*N* = 13)

| **Category** |  |  |  |  |  | ***n*** | **%** |  | **Example** |  | **Description** |
| --- | --- | --- | --- | --- | --- | --- | --- | --- | --- | --- | --- |
| **Time spent on daily surveys** | | | | |  | 13 | 100,0 |  |  |  | This category encompasses statements estimating the time required to complete the daily surveys. *Statements evaluating the length—such as whether it was too long or too short—were coded under 'Duration of the study'.* |
| **Feasibility** | | | | |  |  |  |  |  |  |  |
|  | Habituation | | | |  | 12 | 92,3 |  | At the beginning, of course, you had to find your way around, but after the 3rd/4th survey you knew the structure of the questions and it was much easier to integrate them into your everyday life because you already knew what you had to answer |  | Statements indicating that, over time, it became easier and faster to complete the daily surveys. |
|  | Duration of the study | | | |  |  |  |  |  |  |  |
|  |  | Length of the individual surveys (adequate) | | |  | 12 | 92,3 |  | Yes, and that was easy to accommodate, because the time periods were quite short |  | This category encompasses all statements concerning the feasibility of the time required to respond to individual survey time points. |
|  |  | Frequency of daily surveys | | |  |  |  |  |  |  | This category includes all statements concerning the frequency of the daily surveys, specifically the evaluation of their occurrence four times a day. Statements were classified as either adequate, too frequent, or too infrequent. Suggestions for a different survey frequency was categorised in a separate sub-category. |
|  |  |  | Adequate | |  | 10 | 76,9 |  | I found the number of questions completely okay |  |  |
|  |  |  | Too infrequent | |  | 1 | 7,7 |  | I: How did you find the number of surveys per day? P: Doable, so it wasn't too much, rather a little too little |  |  |
|  |  |  | Too frequent | |  | 2 | 15,4 |  | I think four times is too many |  |  |
|  |  |  | Suggestions | |  | 3 | 23,1 |  |  |  |  |
|  |  | Study duration | | |  |  |  |  |  |  | This category includes all statements related to the duration of the study (i.e., 7 days). Statements were coded as the 7-day study period being adequate, too short, or too long. |
|  |  |  | Adequate | |  | 5 | 38,5 |  | So a week was still just about within the limit, I would say |  |  |
|  |  |  | Too short | |  | 7 | 53,8 |  | I could have imagined it being longer. So I could have imagined two weeks being better |  |  |
|  |  |  | Too long | |  | 1 | 7,7 |  | So for me less would have made sense (...) 3 or 4 days |  |  |
|  | Survey time points | | | |  |  |  |  |  |  |  |
|  |  | General | | |  |  |  |  |  |  | This category covers statements discussing the feasibility of the survey time points without referring to any specific one. The statements were classified as high, moderate, or low feasibility. |
|  |  |  | High feasibility | |  | 3 | 23,1 |  | Roughly speaking, the times were okay |  |  |
|  |  |  | Moderate feasibility | |  | 1 | 7,7 |  | Mostly, there's always one that doesn't fit |  |  |
|  |  |  | Low feasibility | |  | 7 | 53,8 |  | It wasn't always suitable, but there would never have been a time when I would have said it would have been better. It's just difficult, depending on employment and appointments |  |  |
|  |  | Specific time points | | |  |  |  |  |  |  | This category includes all statements regarding the feasibility of specific survey time points (i.e., 7:30, 12:00, 16:30, and 21:00). Each time point was categorized into sub-categories, with feasibility rated as either high, moderate (manageable but sometimes also presented challenges), or low. |
|  |  |  | 7:30 | |  |  |  |  |  |  |  |
|  |  |  |  | High feasibility |  | 4 | 30,8 |  | The morning one (...) the time was actually always great, I found |  |  |
|  |  |  |  | Moderate feasibility |  | 3 | 23,1 |  | 7.30 am is also good during the week, on the weekend, well [shrugs], I think I skipped one day [laughs], slept longer that day. But otherwise I'm usually awake by 7.30 am |  |  |
|  |  |  |  | Low feasibility |  | 6 | 46,2 |  | So I found the 7.30 a.m. survey (...) very, very challenging (...) because most of the time it's really the children's drop-off time. So if you have school-age children [laughs] or have to take them to the nursery, it was always a bit difficult to fit that in somehow. And because you were then under a lot of time pressure(…) because you were also travelling to work afterwards |  |  |
|  |  |  | 12:00 | |  |  |  |  |  |  |  |
|  |  |  |  | High feasibility |  | 5 | 38,5 |  | I: There was the 12 o'clock survey?, P: Mhm [nods]. That actually always worked (...) because that's the time of the lunch break |  |  |
|  |  |  |  | Moderate feasibility |  | 4 | 30,8 |  | 12:00 is sometimes a bit hard to do when you have a meeting, at 12:00 you usually have lunch, but that doesn't always work out |  |  |
|  |  |  |  | Low feasibility |  | 2 | 15,4 |  | I: Then we still had 12 o'clock?, P: Rather difficult. (...) Because of [the childrens'] nap time |  |  |
|  |  |  | 16:30 | |  |  |  |  |  |  |  |
|  |  |  |  | High feasibility |  | 4 | 30,8 |  | And 4.30 pm (...) actually works for me on most working days |  |  |
|  |  |  |  | Moderate feasibility |  | 4 | 30,8 |  | Well it was really still feasible, I'd say, it was more like certain individual days, no, where I was for example travelling in the car at the time or something like that, no, it was difficult then, and I think once or twice I definitely missed the period at those times because of that |  |  |
|  |  |  |  | Low feasibility |  | 2 | 15,4 |  | P1: It doesn't really fit into everyday life (...) in the afternoon, as I said, you talk to your child, your partner, you have errands to run, no idea what you have to do in life and then you don't just take the time to say, I need 5 minutes here; P2: There were some times that were difficult to realise, especially 16:30, which is the end of the working day |  |  |
|  |  |  | 21:00 | |  |  |  |  |  |  |  |
|  |  |  |  | High feasibility |  | 8 | 61,5 |  | 9 p.m. is great, it's not a problem, that's when everything is done, the children are asleep, so I have time [laughs] |  |  |
|  |  |  |  | Moderate feasibility |  | 2 | 15,4 |  | 9 p.m. tended to be difficult (...) If [she/he] in bed, the time is good, if [she/he] not yet in bed, the time is unfavourable at best, because then I'm about to put the child to bed |  |  |
|  |  |  |  | Low feasibility |  | 1 | 7,7 |  | And actually for us, we have such an early rising child, 9 pm is quite late. It's always like that, at half past eight, oh, stay awake for another half an hour |  |  |
|  |  |  | Alternatives | |  | 8 | 61,5 |  |  |  | Statements in which participants suggested alternatives for particular time points. |
|  | Response window (30 minute buffer) | | | |  |  |  |  |  |  | Statements evaluating the feasibility of the 30-minute window at each time point for completing the survey were categorized into three groups: adequate, too short, and mixed. |
|  |  | Adequate | | |  | 9 | 69,2 |  | So I thought the buffer [grace period] was good, because I got caught twice in the car (...) So it makes sense, because you have moments when you can't react very well right now |  |  |
|  |  | Too short | | |  | 2 | 15,4 |  | I think it's easier to push something in an hour, but in half an hour it's often difficult, especially if you're at something or on the move until you're back again. You can do a lot in an hour, but I think half an hour is too short |  |  |
|  |  | Mixed | | |  | 1 | 7,7 |  | With the half hour (...) it was already relatively difficult at certain times |  |  |
|  | Miscellaneous | | | |  |  |  |  |  |  |  |
|  |  | Monetary incentive | | |  | 13 | 100,0 |  | I think that's appropriate. So I would have done it without |  | Statements regarding the incentive for participating in the study and the assessment that it was appropriate in relation to the effort required to participate in the study. |
|  |  | Psychological support | | |  | 1 | 7,7 |  | So if, for example, I had always said in this survey that I'm in a really bad way, I realise that I don't really have my life under control, it's of course nice that I realise that. But the fact is that even if I realise that, I have very little chance of getting help. (...) If everything is fine now, then yes, it doesn't matter, but for those who really - where you continuously realise, wow, there's someone here who has really said in every survey that they're in a really bad way, then of course it's a bit unfortunate for them, because they might then realise it even more |  | Statements addressing psychological support in connection with this or similar studies. |
|  |  | Suggestions for improving the study | | |  | 11 | 84,6 |  |  |  | Statements in which participants proposed alternative study designs. *Suggestions related to specific time points are coded separately in Survey time points - specific time points - alternatives. Suggestions to improve the app are coded separately under technical aspects..* |
| **EMA Measurement** | | | | |  |  |  |  |  |  |  |
|  | General comprehension of the daily questions | | | |  |  |  |  |  |  | Any statements concerning the overall comprehension of the survey questions, which were classified into the categories: understandable and some problems. *Statements regarding time-use were coded under 'Time-use measurement via EMA' while those referring to well-being or stress were coded under 'Measurement of well-being & stress via EMA'.* |
|  |  | Understandable | | |  | 8 | 61,5 |  | I: Were the questions formulated clearly for you? P: Yes. |  |  |
|  |  | Some problems | | |  | 4 | 30,8 |  | I: Were the questions formulated clearly?, P: Generally, yes. There was a question somewhere [which was] somehow strange. |  |  |
|  | Measurement of well-being & stress via EMA | | | |  |  |  |  |  |  | This category contains statements related to whether the measurement of well-being and stress via EMA was an adequate reflection of their personal well-being and stress. Statements were classified into the categories adequate, mixed, and not adequate. |
|  |  | Adequate | | |  | 6 | 46,2 |  | Yes, the questions were also multi-, multi-faceted, if I can call it that, so whether you're nervous or depressed at the moment, those are two different things. And I actually felt well categorised by the questions - what my my mood is like at the moment or my stress level. |  |  |
|  |  | Mixed | | |  | 4 | 30,8 |  | I: Did you have the feeling that your well-being or perceived stress was adequately measured throughout the day? P: Yes [hesitantly]. Yes, so it was measured, yes, but I didn't experience any major fluctuations [laughs]. That's why I perhaps didn't perceive it as a real measurement |  |  |
|  |  | Not adequate | | |  | 3 | 23,1 |  | Because the questions and the answers were worded too generally and too broadly to be able to capture selective fluctuations |  |  |
|  | Time-use measurement via EMA | | | |  |  |  |  |  |  |  |
|  |  | Measurement of daily activities | | |  |  |  |  |  |  | Statements were categorized in this category when they referred to the measurement of current activities or the sequence of activities between time points. They were further classified based on whether the measurement of the current activity or the sequence in between time-points accurately represented their overall daily activities or routines either adequately or not adequately. |
|  |  |  | Current activitiy | |  |  |  |  |  |  |  |
|  |  |  |  | Adequate |  | 3 | 23,1 |  | I would say so, yes. [nods] So yes, they actually basically reflect what I do throughout the day |  |  |
|  |  |  |  | Not adequate |  | 10 | 76,9 |  | Nah [shaking head]. It varies depending on, well, depending on the day, I don't work every day. Well, I'm not employed every day [smiles] and (...) that's why it changes. Or sometimes I only work half days and then I do something different in the mornings than in the afternoons. (...) And that's why, if you only looked at the moment, it wouldn't reflect everything that happens during the day |  |  |
|  |  |  | Sequence in between time-points | |  |  |  |  |  |  |  |
|  |  |  |  | Adequate |  | 10 | 76,9 |  | I: And if you think the other way round, just, not the momentary query, but just the in-between? (...) Do you think that would be a reflection of your entire everyday life?, P: More likely |  |  |
|  |  |  |  | Not adequate |  | 3 | 23,1 |  | I: And if you now think the other way round, i.e. only about the activities between the interviews, would you say that they would reflect your entire everyday life?; P: No |  |  |
|  |  | Assignment of activities to the categories | | |  |  |  |  |  |  | Statements indicating whether participants were certain about how to categorize one or more of their daily activities within the provided activity groups. Statements were grouped into clear and some uncertainty (if participants provided conflicting statements that could not be definitively classified as either clear or unclear). |
|  |  |  | Clear | |  | 6 | 46,2 |  | No, I think it showed everything I would have needed |  |  |
|  |  |  | Some uncertainty | |  | 7 | 53,8 |  | Something like physical activity, I don't know if that was included, I categorised it as a leisure time/hobby, so to speak, where I thought about whether it wasn't also a bit of self-care, but yes. So sporting activity/sports, whether you take that extra, because that could perhaps also be stress-relieving |  |  |
|  |  | Reporting the sequence of activities | | |  | 9 | 69,2 |  | I think what I also didn't find intuitive was these different activities, I think they were always asked about them individually one after the other. And that was somehow (...) a bit (...) when I think about it, I think the first time they asked me what I was doing now. And then there were also the option of multiple answers. So I don't know, I just found it a bit confusing, perhaps really. Because I think the second time I was asked what I had already entered. In any case, I didn't find it very clear in retrospect. |  | Statements reflecting confusion about reporting the sequence of daily activities between survey time points are categorized here. This confusion could involve uncertainty about first reporting the current activity followed by the sequence since the last time point, or about the instruction to report multiple activities between time points, when there was no clear sequence to report due to minimal changes in activities since the previous time point. |
|  |  | Multiple activities | | |  | 5 | 38,5 |  | So it's also difficult, especially because you sometimes do several things at the same time. I have the feeling that I didn't find it easy to visualise (...) when, for example, I was doing housework and childcare at the same time, but started at different times, then I actually ticked the box saying I was doing both at the same time. But I didn't actually start at the same time. (...) So now it's an ‘and’ question or an ‘or’ question. |  | Any statements addressing the benefits or challenges of reporting multiple activities occurring simultaneously, such as watching TV and providing childcare, which do not necessarily share the exact same start and end times. |
|  |  | Time entry | | |  | 4 | 30,8 |  | This time input really irritated me, actually. |  | Any statements about the method of recording the start or end times of daily activities, whether criticizing or praising the approach. |
|  |  | Ability to Recall Daily Activities | | |  | 12 | 92,3 |  | I could remember it well. |  | Statements regarding the high ability to recall daily activities between survey time points. |
| *Note*. Multiple text segments for a participant were counted only once per category, i.e., if a participant made conflicting statements regarding time point 7:30 these were coded as moderately feasibility and NOT separately for high and low feasibility. Certain categories were only classed as high feasibility (e.g., study length), because no statements with other levels of feasibility were made by participants. A mixed-method study on compliance and feasibility of ecological momentary assessment surveys examining daily well-being and time-use in a German parent sample. | | | | | | | | | | | |
