# Supplementary material for: Ecological Momentary Assessment of Parental Well-Being and Time Use: Mixed Methods Compliance and Feasibility Study
Source: JMIR Form Res. 2025 Apr 23;9:e67451. doi: 10.2196/67451 (PMC12059499; doi:10.2196/67451)
Supplement: Multimedia Appendix 6 [file formative_v9i1e67451_app6.docx]

**Multimedia appendix 6.** Technical aspects

Most interviewees assessed the app as feasible for the study and nearly all mentioned that it was user friendly. Yet, a number of technical issues were raised at follow-up. For instance, issues with compatibility of the app were mentioned in both the questionnaires and the interviews. This included the need for an alternative download instruction due to older or newer Android versions on the personal smartphone, display issues due to compatibility with the Android system, and criticism regarding the app not being available to Apple smartphone users at the time. Interviewees highlighted the reminders to the daily surveys as useful, however in both the follow-up questionnaires and interviews, participants also reported that not all the reminders to the daily surveys were displayed; either failing randomly, at specific time-points (e.g., 9pm), or starting a day later than planned. Another issue mentioned in both the follow-up questionnaires and interviews was that the snooze function to answer the survey within the 30 minute-period did not work automatically and instead needed to be activated manually by pressing a ‘snooze-button’. One interviewee also mentioned that they received surveys double. None of the interviewees had any concerns regarding data protection. Interviewees appreciated the “quick” and “good” support or requested better technical support during the study period. In the follow-up questionnaires and interviews participants made a number of suggestions for the app-improvement, including: a different app-design (e.g., regarding layout or colour scheme), back button when filling in the survey, notification when survey period finished, and an automatic snooze function.

**Table 1.** Descriptive results regarding technical aspects

|  |  |  |  |  |
| --- | --- | --- | --- | --- |
|  | ***n*** | ***miss.*** | ***M*** | ***SD*** |
| On a scale of 1 (bad) - 10 (good), how ... |  |  |  |  |
| ...well did the mobile application/app (movisensXS) work? | 67 | 0 | 7.01 | 2.520 |
| ... intuitive was the use of the mobile application/app (movisensXS)? | 67 | 0 | 7.04 | 2.325 |
|  |  |  |  |  |
| How easy is it to learn how to use the mobile application/app (movisensXS)?  *(1) no explanation, confusing & complicated –*  *(5) intuitive & user friendly* | 67 | 0 | 3.91 | 0.965 |
|  |  |  |  |  |
| In my opinion... |  |  |  |  |
| *(1) Strongly disagree - (5) Strongly agree* |  |  |  |  |
| ...it was boring to work with the mobile application/app (movisensXS). | 66 | 1 | 2.32 | 1.205 |
| ...the mobile application/app (movisensXS) was easy to use. | 66 | 1 | 4.15 | 1.026 |
| *Note*. miss. = missing. | | | | |

**Table 2.** Categories concerning technical aspects (follow-up questionnaires) (*n* = 57)

| **Category** | |  | ***n*** | **%** |  | **Example** | **Description** |
| --- | --- | --- | --- | --- | --- | --- | --- |
| App (high feasibility) | |  | 6 | 10,5 |  | The app is good | Statements made that expressed that the app was good and functional for the purposes of the survey. |
| Problems | |  |  |  |  |  | Statements addressing technical issues with the app were categorized here. These were divided into suggestions to improve the app design, problems with the operating system of the smartphone, survey reminders, the snooze function and the compatibility of the app (e.g., display issues). |
|  | App-Design (suggestions for improvement) |  | 14 | 24,6 |  |  |  |
|  | Operating system |  | 4 | 7,0 |  | Availability of the app on iOS |  |
|  | Survey reminders |  | 12 | 21,1 |  | And sometimes I didn't get the notification at [the] survey time because [the app] didn't display the survey. |  |
|  | Snooze function |  | 9 | 15,8 |  | Snooze mode generally did not work |  |

*Note*. Multiple text segments for a participant were counted only once per category, i.e., if a participant made conflicting statements regarding time point 7:30 these were coded as moderately feasibility and NOT separately for high and low feasibility. Certain categories were only classed as high feasibility (e.g., study length), because no statements with other levels of feasibility were made by participants. Out of 63 participants who completed the follow-up questionnaires, only 57 participants responded to the open-ended questions regarding feasibility.

**Table 3.** Categories concerning technical aspects (interviews) (*n*  = 13)

| **Category** | | | |  | ***n*** | **%** |  | **Example** | **Description** |
| --- | --- | --- | --- | --- | --- | --- | --- | --- | --- |
| **App-Design** | | | |  |  |  |  |  |  |
|  | Intuitive | | |  | 12 | 92,3 |  | The app is super user-friendly. | Statements in which respondents rated the intuitiveness of the app design. |
|  | App design was functional | | |  | 13 | 100,0 |  |  | Statements in which respondents rated the functionality of the app design. Statements specifically regarding the survey reminder were coded in a sub-category. |
|  |  | Survey reminders | |  | 4 | 30,8 |  | the option of being reminded directly via the app (…) because it can happen in everyday life that you don't remember the different times, I think that's quite well done |  |
|  | Improvement of the App | | |  | 10 | 76,9 |  | Maybe play with the colours a bit. So this grey black and white is somehow pre-medieval | Statements containing ideas for improving the design or layout of the app. *Statements were coded here if they were not related to the study design and exclusively concerned optimising the app.* |
| **Problems** | | | |  |  |  |  |  | Statements addressing technical issues with the app were categorized here. These were divided into problems with the survey reminders, the snooze function and a request for an automatic snooze feature, issues with survey notifications, difficulties with downloading the app, and challenges in completing the survey. |
|  | Survey reminder | | |  |  |  |  |  |  |
|  |  | Problems with the survey snooze function | |  | 6 | 46,2 |  | If I had known then that I could postpone them, but that I had to react immediately to postpone them, then I would have done so. But I let it ring briefly and then it was already over and I couldn't access them at all. |  |
|  |  |  | Wish for an automatic snooze function |  | 3 | 23,1 |  | I would have wished(...) because I had always hoped that if he didn't ring the first time, it might automatically ring again after half an hour. |  |
|  |  | Survey reminder | |  | 5 | 46,2 |  | So during the course of the study I had a minor problem (...) and that was, I think, I don't remember exactly, from day 5 or 6 the 12 o'clock time point no longer appeared |  |
|  | App download | | |  | 7 | 53,8 |  | I already had problems with the download because I couldn't install it on my smartphone |  |
|  | Filling out the daily surveys | | |  | 2 | 15,4 |  | Some things were actually surveyed twice |  |
| **Technical support** | | | |  | 6 | 46,2 |  | P1: I actually tried to call about this, but unfortunately I often didn't get through to anyone; P2: If anything was unclear, I wrote to you and then you wrote back to me directly, so from there. (NARI686) | Statements related to technical support were categorized here, either highlighting praise for the support received or expressing a need for improved technical assistance. |
| **Data protection (no concerns)** | | | |  | 13 | 100,0 |  | No, I had no concerns because I assumed that the personal data collected there would be handled according to a specific regulation. I had no concerns about using the app. | Statements expressing (no) concerns about the data privacy of study information were coded in this category. |

*Note*. Multiple text segments for a participant were counted only once per category, i.e., if a participant made conflicting statements regarding time point 7:30 these were coded as moderately feasibility and NOT separately for high and low feasibility. Certain categories were only classed as high feasibility (e.g., study length), because no statements with other levels of feasibility were made by participants.
