# Supplementary material for: Ecological Momentary Assessment of Parental Well-Being and Time Use: Mixed Methods Compliance and Feasibility Study
Source: JMIR Form Res. 2025 Apr 23;9:e67451. doi: 10.2196/67451 (PMC12059499; doi:10.2196/67451)
Supplement: Multimedia Appendix 7 [file formative_v9i1e67451_app7.docx]

**Multimedia appendix 7.** Frequency and description of the themes from the baseline questionnaires (*N* = 74)

| **Category** |  |  |  |  |  | ***n*** | **%** |  | **Example** |  | **Description** |
| --- | --- | --- | --- | --- | --- | --- | --- | --- | --- | --- | --- |
| **Feasibility** | | | | |  |  |  |  |  |  |  |
|  | General feasibility | | | |  |  |  |  |  |  | This category includes all statements related to the overall feasibility of the study design and integration into participants' everyday life. It encompasses evaluations of the study's ‘do-ability’ in general. Statements in this category are classified as high feasibility (possible to accommodate the daily surveys and no barriers mentioned) and moderate feasibility (some concerns regarding at least one part of the survey design were mentioned). *If statements included high, moderate, or low feasibility of specific aspects of the survey design, this was also coded elsewhere (i.e., survey length, survey time points). Statements that are exclusively related to the survey app and not to the study design are coded elsewhere under 'technical aspects'.* |
|  |  | High feasibility | | |  | 20 | 27,8 |  | It is doable |  |  |
|  |  | Moderate feasibility | | |  | 51 | 70,8 |  | Minor concerns(...) Otherwise certainly feasible |  |  |
|  | Study duration (high feasibility) | | | |  | 34 | 47,2 |  | P1: 7 days is the optimal period for the survey; P2: 5 minutes should be ok |  | Statements that highlighted the high feasibility of the survey duration were coded here, that is either mention the seven day survey period or the time to complete individual surveys. |
|  | Concerns | | | |  |  |  |  |  |  | These sub-categories encompass all statements expressing concerns or anxieties about the study design. The statements were classified into three groups: no concerns, concerns related to time management (such as difficulties responding to the survey at specific times or integrating the study design into daily routines), and concerns about potential app malfunctions. |
|  |  | No concerns | | |  | 53 | 73,6 |  | I: Do you have any other thoughts or concerns about the procedure?, P: No |  |  |
|  |  | Time management | | |  | 5 | 6,9 |  | My main concern is that I won't be able to ensure that I have enough time for some surveys, as there is often no time buffer due to my job and three school children with their appointments. |  |  |
|  |  | App | | |  | 1 | 1,4 |  | I hope that the app and the integration of the study will function |  |  |
|  | Excitement and enthusiasm | | | |  | 9 | 12,5 |  | I am excited |  | Statements in which participants expressed their excitement and enthusiasm about the start of the study and their participation in it. |
|  | Survey time points | | | |  |  |  |  |  |  |  |
|  |  | Time points in general (low feasibility) | | |  | 6 | 8,3 |  | Fixed time points could become difficult |  | This category includes all statements regarding the feasibility of the survey times (7:30, 12:00, 16:30, and 21:00). Each time point was organized into sub-categories, with feasibility rated as either high, moderate or low. Statements are coded as moderately feasible when participants provided mixed feedback, noting that it may be manageable but can also present challenges. |
|  |  | 7:30 | | |  |  |  |  |  |  |  |
|  |  |  | High feasibility | |  | 3 | 4,2 |  | I don't think it's a problem in the morning |  |  |
|  |  |  | Low feasibility | |  | 29 | 40,3 |  | The one at 7.30 is bad. Because that's when I have to get the kids ready |  |  |
|  |  | 12:00 | | |  |  |  |  |  |  |  |
|  |  |  | High feasibility | |  | 11 | 15,3 |  | For me, 12 o'clock (...) fits in well |  |  |
|  |  |  | Moderate feasibility | |  | 2 | 2,8 |  | On the weekend, the lunchtime survey coincides with my daughter's nap. |  |  |
|  |  |  | Low feasibility | |  | 5 | 6,9 |  | 12 o'clock is difficult because I (...) work at 12 o'clock. |  |  |
|  |  | 16:30 | | |  |  |  |  |  |  |  |
|  |  |  | High feasibility | |  | 4 | 5,6 |  | 16.30 (...) should work well |  |  |
|  |  |  | Moderate feasibility | |  | 1 | 1,4 |  | [16:30 is] dependent on the day and schedule |  |  |
|  |  |  | Low feasibility | |  | 12 | 16,7 |  | The survey in the afternoon will be a bit more difficult, as this is the “peak time” in family life |  |  |
|  |  | 21:00 | | |  |  |  |  |  |  |  |
|  |  |  | High feasibility | |  | 12 | 16,7 |  | I don't think it's a problem in the evening |  |  |
|  |  |  | Low feasibility | |  | 5 | 6,9 |  | That won't be easy (...) sometimes not all the children are asleep at 9 pm. |  |  |
|  |  | Response window (30 minute buffer) | | |  |  |  |  |  |  | When statements addressed the 30-minute window for completing the survey at each time point, they were classified in this category. They were coded as either too short or adequate length. |
|  |  |  | Too short | |  | 4 | 5,6 |  | A time window of 60 minutes rather than just 30 minutes would have been more ideal. |  |  |
|  |  |  | Adequate length | |  | 3 | 4,2 |  | The time window of 30 minutes also seems appropriate to me. |  |  |
| **EMA Measurement** | | | | |  |  |  |  |  |  |  |
|  | Anticipated Ability to Recall Daily Activities | | | |  |  |  |  |  |  | Statements regarding the anticipated ability to recall daily activities between survey time points were categorized as follows: good (confidence in recalling all activities), moderately good, and not good (believing it would be challenging to remember all activities). |
|  |  | Good | | |  | 60 | 83,3 |  | With some deliberation it will work |  |  |
|  |  | Moderate | | |  | 7 | 9,7 |  | Sometimes I'm sure I will forget something |  |  |
|  |  | Not good | | |  | 1 | 1,4 |  | It will certainly be difficult for me |  |  |
| *Note*. Multiple text segments for a participant were counted only once per category, i.e., if a participant made conflicting statements regarding time point 7:30 these were coded as moderately feasibility and NOT separately for high and low feasibility. Certain categories were only classed as high feasibility (e.g., study length), because no statements with other levels of feasibility were made by participants. A mixed-method study on compliance and feasibility of ecological momentary assessment surveys examining daily well-being and time-use in a German parent sample. | | | | | | | | | | | |
