# Supplementary material for: Ecological Momentary Assessment of Parental Well-Being and Time Use: Mixed Methods Compliance and Feasibility Study
Source: JMIR Form Res. 2025 Apr 23;9:e67451. doi: 10.2196/67451 (PMC12059499; doi:10.2196/67451)
Supplement: Multimedia Appendix 8 [file formative_v9i1e67451_app8.docx]

**Multimedia appendix 8.** Frequency and description of the themes from the follow-up questionnaires (*N* = 57)

| **Category** | | | | |  | ***n*** | ***%*** |  | **Example** |  | **Description** |
| --- | --- | --- | --- | --- | --- | --- | --- | --- | --- | --- | --- |
| Feasibility | | | | |  |  |  |  |  |  |  |
|  | Habituation | | | |  | 3 | 5,3 |  | During the survey, I experienced a certain habituation effect due to the consistency |  | Statements indicating that, over time, it became easier and faster to complete the daily surveys. |
|  | Frequency of daily surveys | | | |  |  |  |  |  |  | This category includes all statements concerning the frequency of the daily surveys, specifically the evaluation of their occurrence four times a day. Statements were classified as either too frequent or too infrequent. |
|  |  | Too frequent | | |  | 5 | 8,8 |  | Survey only once/twice a day |  |  |
|  |  | Too infrequent | | |  | 1 | 1,8 |  | Increase the number of alarms per survey time by 2 |  |  |
|  | Study duration | | | |  |  |  |  |  |  | This category includes all statements related to the duration of the study (i.e., 7 days). Statements were coded as the 7-day study period being too short, adequate, longer but less time points, or too long. |
|  |  | Too short | | |  | 11 | 19,3 |  | 7 days are almost too short. Four of the seven days were not typical for us. |  |  |
|  |  | Adequate | | |  | 18 | 31,6 |  | 7 days was okay |  |  |
|  |  | Longer but less time points | | |  | 3 | 5,3 |  | Longer but only once/twice a day |  |  |
|  |  | Too long | | |  | 2 | 3,5 |  | I: I would plan the following trial duration..., P1: 3 days / P2: 5 days |  |  |
|  | Survey time points | | | |  |  |  |  |  |  |  |
|  |  | General | | |  |  |  |  |  |  | This category covers all statements discussing the feasibility of the survey time points without referring to any specific ones. The statements were classified as high or low feasibility. |
|  |  |  | High feasibility | |  | 2 | 3,5 |  | Well chosen time points |  |  |
|  |  |  | Low feasibility | |  | 5 | 8,8 |  | For us, these were always exactly the times when there was a change of activities, which is why it was sometimes difficult |  |  |
|  |  | Specific time points | | |  |  |  |  |  |  | This category includes all statements regarding the feasibility of specific survey times (7:30, 12:00, 16:30, and 21:00). With statements categorised into sub-categories of high, moderate or low feasibility. Where participants offered alternative suggestions for a specific time point this was coded in a separate sub-category. |
|  |  |  | 7:30 | |  |  |  |  |  |  |  |
|  |  |  |  | Low feasibility |  | 28 | 49,1 |  | 7:30 is a critical time on the way to nursery and there was sometimes no room for the survey |  |  |
|  |  |  |  | Suggestion |  | 12 | 21,1 |  | P1: 7:30 possibly at 8:00 or 9:00, as drop-off time to the nursery is until 8:00; P2: 6:30 instead of 7:30 |  |  |
|  |  |  | 12:00 | |  |  |  |  |  |  |  |
|  |  |  |  | High feasibility |  | 1 | 1,8 |  | Appointment at 12:00 noon was (…) a good time to respond due to lunch break and/or time without childcare or with organization/household/self-care, which could be briefly interrupted for the survey. |  |  |
|  |  |  |  | Moderate feasibility |  |  |  |  | The time point at 12 noon is normally very convenient, but unfortunately I had appointments on some days that I don't normally have at this time. |  |  |
|  |  |  |  | Low feasibility |  | 4 | 7,0 |  | 12h in the middle of work |  |  |
|  |  |  |  | Suggestion |  | 3 | 5,3 |  | P1: 12 o'clock appointment would have been better at 11:30; P2: 2 p.m. after-work with all its obligations has not yet started |  |  |
|  |  |  | 16:30 | |  |  |  |  |  |  |  |
|  |  |  |  | High feasibility |  | 1 | 1,8 |  | The survey time at 16:30 was excellent for me |  |  |
|  |  |  |  | Low feasibility |  | 4 | 7,0 |  | 16:30 commute to school and work |  |  |
|  |  |  |  | Suggestion |  | 4 | 7,0 |  | P1: 16:30 to 17:00; P2: 17:30 instead of 16:30 |  |  |
|  |  |  | 21:00 | |  |  |  |  |  |  |  |
|  |  |  |  | High feasibility |  | 2 | 3,5 |  | The survey time at 9 p.m. is very good |  |  |
|  |  |  |  | Low feasibility |  | 11 | 19,3 |  | 21:00 was sometimes too late for me |  |  |
|  |  |  |  | Suggestion |  | 1 | 1,8 |  | P1: 9 p.m. was too late for me; P2: 9 p.m.: the children are not always ready for bed at this time. |  |  |
|  |  | Customizable time points for individual needs | | |  | 6 | 10,5 |  | A little more flexibility in the survey time would relax the respondents |  | Statements expressing a desire for more flexibility regarding the time points, that is where participants indicated a preference for being able to select time points that align with their individual routines and lifestyles. |
|  |  | Response window (30 minute buffer) (too short) | | |  | 4 | 7,0 |  | I would find it easier to manage a 1-hour buffer |  | When statements described the 30-minute window for completing the survey at each time point as too short. |
| EMA Measurement | | | | |  |  |  |  |  |  |  |
|  | Challenges in Survey Comprehension and EMA Measurement | | | |  | 8 | 14,0 |  | The questions should be formulated more precisely |  | Statements expressing that the survey wording was generally hard to comprehend or highlighting other challenges with the EMA measurement. *Challenges specifically related to time use measurement are coded separately.* |
|  | Time use | | | |  |  |  |  |  |  |  |
|  |  | Assignment of activities to the categories unclear | | |  | 3 | 5,3 |  | Unfortunately, I couldn't differentiate the activities because childcare as an activity was more or less always active and the main activity besides work. Even if I watched TV with my child, for example, I don't do this as a hobby but under the keyword childcare. |  | Statements indicating that participants were uncertain about how to categorize one or more of their daily activities within the provided activity groups. |
|  |  | Sequence of activities | | |  | 16 | 28,1 |  | The sequence of questions did not move chronologically backwards or forwards, but from the current activity to the last activity after the last survey and then back to the current activity. It was therefore difficult to differentiate between the times here |  | Statements regarding the recall and reporting of the sequence of daily activities between survey time points. |
|  |  |  | Overview of daily activities | |  | 7 | 12,3 |  | The query of the duration of the activities could be done better with an interactive Gannt chart or an interactive clock |  | Any statements from participants expressing a desire for a (visual) overview of their daily activities. |
|  |  | Multiple activities | | |  | 5 | 8,8 |  | The activity query for more than one activity is very confusing |  | Any statements addressing the benefits or challenges of reporting multiple activities occurring simultaneously, such as watching TV and providing childcare, which do not necessarily share the exact same start and end times. |
|  |  | Time entry | | |  | 9 | 15,8 |  | The time entry was extremely misleading and not very intuitive (time or duration?) |  | Any statements about the method of recording the start or end times of daily activities, whether criticizing or praising the approach. |
| *Note*. Multiple text segments for a participant were counted only once per category, i.e., if a participant made conflicting statements regarding time point 7:30 these were coded as moderately feasibility and NOT separately for high and low feasibility. Certain categories were only classed as high feasibility (e.g., study length), because no statements with other levels of feasibility were made by participants. Out of 63 participants who completed the follow-up questionnaires, only 57 participants responded to the open-ended questions regarding feasibility. A mixed-method study on compliance and feasibility of ecological momentary assessment surveys examining daily well-being and time-use in a German parent sample. | | | | | | | | | | | |
